# The citrullinated/native index of autoantibodies against hnRNP-DL predicts an individual “window of treatment success” in RA patients

**DOI:** 10.1186/s13075-021-02603-x

**Published:** 2021-09-14

**Authors:** Bianka Marklein, Madeleine Jenning, Zoltán Konthur, Thomas Häupl, Franziska Welzel, Ute Nonhoff, Sylvia Krobitsch, Debbie M. Mulder, Marije I. Koenders, Vijay Joshua, Andrew P. Cope, Mark J. Shlomchik, Hans-Joachim Anders, Gerd R. Burmester, Aase Hensvold, Anca I. Catrina, Johan Rönnelid, Günter Steiner, Karl Skriner

**Affiliations:** 1 Department of Rheumatology and Clinical Immunology, Charité — Universitätsmedizin Berlin, Charite Campus Mitte, Rheumatologisches Forschungslabor - AG Skriner, Chariteplatz 1 (intern Virchowweg 11, 5.OG, R011), 10117 Berlin, Germany; 2 German Rheumatism Research Centre, Leibniz Institute, 10117 Berlin, Germany; 3 Max Planck Institute for Molecular Genetics, Berlin, Germany; 4 Max Planck Institute of Colloids and Interfaces, Potsdam, Germany; 5 Department of Analytical Chemistry (Dpt.1), Bundesanstalt für Materialforschung und-prüfung (BAM), Berlin, Germany; 6 Department of Experimental Rheumatology, Radboud University Medical Center, Nijmegen, The Netherlands; 7 Division of Rheumatology, Department of Medicine Solna, Karolinska Institutet, Karolinska University Hospital, Stockholm, Sweden; 8 Centre for Rheumatic Diseases, School of Immunology and Microbial Sciences, Faculty of Life Sciences and Medicine, King’s College London, London, UK; 9 Department of Immunology, University of Pittsburgh School of Medicine, Pittsburgh, PA USA; 10 Medical Clinic and Policlinic IV, Nephrological Center, Ludwig-Maximilian-University Hospital, Munich, Germany; 11 Academic Specialist Center, Center for Rheumatology, Stockholm Health Region, Stockholm, Sweden; 12 Department of Immunology, Genetics and Pathology, Uppsala University, Uppsala, Sweden; 13 Division of Rheumatology, Medical University of Vienna, Vienna, Austria; 14 Ludwig Boltzmann Cluster for Arthritis and Rehabilitation, Vienna, Austria

**Keywords:** Rheumatoid arthritis, ACPA, Anti-CCP, Rheumatoid factor, Shared epitope, Systemic lupus erythematosus, Autoantigens, Treatment

## Abstract

**Background:**

There is a need for biomarker to identify patients “at risk” for rheumatoid arthritis (risk-RA) and to better predict the therapeutic response and in this study we tested the hypothesis that novel native and citrullinated heterogeneous nuclear ribonucleoprotein (hnRNP)-DL autoantibodies could be possible biomarkers.

**Methods:**

Using protein macroarray and ELISA, epitope recognition against hnRNP-DL was analysed in sera from different developed RA disease and diagnosed SLE patients. Toll-like receptor (TLR) 7/9 and myeloid differentiation primary response gene 88 (MyD88)-dependency were studied in sera from murine disease models. HnRNP-DL expression in cultivated cells and synovial tissue was analysed by indirect immunofluorescence, immunoblot and immunohistochemistry.

**Results:**

HnRNP-DL was highly expressed in stress granules, citrullinated in the rheumatoid joint and targeted by autoantibodies either as native or citrullinated proteins in patient subsets with different developed RA disease. Structural citrullination dependent epitopes (SCEs) of hnRNP-DL were detected in 58% of the SLE patients although 98% of these sera were α-CCP-2-negative. To obtain a specific citrullinated signal value, we subtracted the native antibody value from the citrullinated signal. The citrullinated/native index of autoantibodies against hnRNP-DL (CN_DL_-Index) was identified as a new value for an “individual window of treatment success” in early RA and for the detection of RF IgM/α-CCP-2 seronegative RA patients (24–46%). Negative CN_DL_-index was found in SLE patients, risk-RA and early RA cohorts such as EIRA where the majority of these patients are DAS28-responders to methotrexate (MTX) treatment (87%). High positive CN_DL_-values were associated with more severe RA, shared epitope and parenchymal changes in the lung. Specifically, native α-hnRNP-DL is TLR7/9-dependent, associated with pain and ROC analysis revealed an association to initial MTX or etanercept treatment response, especially in seronegative RA patients.

**Conclusion:**

CN_DL_-index defines people at risk to develop RA and the “window of treatment success” thereby closing the sensitivity gap in RA.

**Supplementary Information:**

The online version contains supplementary material available at 10.1186/s13075-021-02603-x.

## Background

More than 20 years ago heterogeneous nuclear ribonucleoprotein (hnRNP) complexes were first described as autoimmune targets [1, 2]. These complexes associate with DNA and RNA and can stimulate Toll-like receptor (TLR) 7 and 9 [3–7]. Antibodies against these structures are characteristic for autoimmune disorders, such as systemic lupus erythematosus (SLE), progressive systemic sclerosis (scleroderma), primary Sjögren’s syndrome, HTLV-1-associated myelopathy/tropical spastic paraparesis (HAM/TSP), multiple sclerosis (MS) and rheumatoid arthritis (RA) as well as for mouse models of lupus and arthritis [8–10].

In RA, the most specific anti-nuclear reactivity is directed against hnRNPs. Most prominent targets are hnRNP-A1 and hnRNP-A2/B1 proteins, which with hnRNP-A3 and hnRNP-A0 proteins form the subgroup of hnRNP-A/B proteins [11–15]. Autoantibodies against hnRNP-A2/B1 (RA33) occur in about 20–40% of RA, SLE and mixed connective tissue disease (MCTD) patients [16]. Autoantibodies to hnRNP-A1 can be found in RA, SLE and MCTD, but probably are cross-reacting α-hnRNP-A2/B1 antibodies [17]. Also, hnRNP-A2/B1 is citrullinated in the rheumatoid joint, and it can be targeted either as a citrullinated and or native protein in distinct subsets of RA patients [18].

Previously, we have described autoantibodies directed to the TNFα regulatory protein hnRNP-D (AUF1) to occur in 33% of SLE, 20% of RA and 17% of MCTD patients [19]. Although predominantly localized in the nucleus, hnRNPs are exported additionally into the cytosol, where they form new autoimmune target structures in stress granules, P-bodies or RNA transport particles [19–21].

The hnRNP-D-like protein (hnRNP-DL) protein, which is also known as JKTBP, is related to the autoantigen hnRNP-D/AUF1. Due to its binding properties and structural features [22], hnRNP-DL,-D and -AB- form the D-subgroup of hnRNPs. These proteins exhibit a modular structure and conserved residues, two adjacent RNA binding domains (RBD) followed by a glycine-rich C-terminal auxiliary domain. However, they are very distinct in each of the unique N-terminal regions [23, 24].

HnRNP-DL acts as a transcription factor [25], participates in metabolism and biogenesis of mRNA [3], is able to shuttle between the nucleus and the cytoplasm and binds both to nuclear and cytoplasmic mRNAs [24], especially when containing AU-rich elements (AREs) as found within the 3′-UTR of many proto-oncogenes and cytokine mRNAs [26, 27]. Up to now, three alternatively spliced hnRNP-DL transcript variants have been described, hnRNP-DL isoform 1–3, whereas proteins only were described for isoform 1 and 2 [23]. Splenocytes from pristane-primed rats restimulated with hnRNPs (-A1,-A2/B1 and -A3) induce a highly inflammatory and erosive arthritis in naïve recipient rats [6]. Furthermore, human TNFα-transgenic mice, which develop a massive erosive inflammatory polyarthritis, generate α-hnRNP autoantibodies [28]. This supports the hypothesis of a pathogenic role of native hnRNPs in erosive arthritis and suggests that autoimmunity to nucleic acid-associated autoantigens has the potential to contribute to RA development [18]. HnRNPs may also induce pro-inflammatory cytokines, relevant for arthritis development in rats, which involve TLR7 and TLR9 but not TLR4 [6].

For α-hnRNP-A2/B1, clinical associations have already been shown for RA severity, with antibodies against the citrullinated protein occurring more frequently in erosive RA and antibodies against the native protein in milder disease [18, 29]. For citrullinated peptides, it has already been shown that the formation of a delta value with the corresponding arginine peptide increased diagnostic sensitivity and indicated association to shared epitope (SE) [30].

In our study, the delta value of ELISA signals was evaluated as a possible biomarker to obtain a new clinical value, as the difference between the α-citrullinated and α-native protein value. hnRNPs were further investigated in the immunopathogenesis of RA, demonstrating the clinical relevance of autoantibodies, for predicting therapeutic success, early parenchymal changes in the lung, and SE in RA. For the first time, structural epitopes resulting from the citrullination process were investigated.

## Material and methods

### Patient sera

A total of 1010 sera were evaluated, including patients with early RA (EIRA cohort *n* = 404), early RA with lung association (LURA cohort *n* = 106), established rheumatoid arthritis (predict cohort *n* = 127), systemic lupus erythematosus (*n* = 89), multiple sclerosis (*n* = 20), reactive arthritis (*n* = 7), scleroderma (*n* = 20), Sjögren’s syndrome (*n* = 20), psoriasis arthritis (*n* = 20), ankylosing spondylitis (*n* = 20), osteoarthritis (*n* = 20), people at risk for developing RA (Risk-RA cohort from Sweden *n* = 62; Risk-RA from Erlangen *n* = 9) and healthy control subjects (*n* = 86). The sera were derived from the serum bank of the 2nd Department of Medicine-Centre of Rheumatic Diseases, Hiezing Hospital (Vienna, Austria), and of the Department of Rheumatology at the Charité Universitätsmedizin (Berlin, Germany). Early RA sera from the Swedish EIRA [31] and LURA [32] cohort and Risk-RA patients were provided by the early arthritis clinic of Karolinska University Hospital in Stockholm, Sweden. Further, we obtained sera from Risk-RA also from the Institute of Rheumatology and Immunology of the University of Erlangen, Germany.

All patients with RA fulfilled the 1987 revised criteria of the American College of Rheumatology [33]. All patients with SLE met the 1982 criteria of the ACR [34], and all patients with MCTD met the criteria described by Alarcon-Segovia and Villarreal [35].

### Mice sera

A total of 153 mice sera were evaluated. SKG Zymosan model (*n* = 16; King’s College, London, England), MRL/lpr (*n* = 20), MRL lpr MyD88^−/−^ (*n* = 20), MRL/lpr TLR7^−/−^ (*n* = 7), MRL/lpr TLR9^−/−^ (*n* = 4), MRL/lpr TLR7/9^−/−^ (*n* = 7, all 5 from Yale University School of Medicine, New Haven), C57BL/6 lpr (*n* = 12), C57BL/6 lpr SIGIRR/TIR8^−/−^ (*n* = 12, both from Medical Policlinics, University Munich), C57BL/6 (*n* = 10), C57BL/6 +R848 (*n* = 10, both from University Hospital of Zurich, Zurich, Switzerland), and Balb/c IL-1Ra^−/−^ (*n* = 35, Radboud University Medical Center 272, Experimental Rheumatology Nijmegen, The Netherlands).

### Cell lines

HEp-2 cell slides were supplied by Generic Assays (ANA HEp-2 plus Kit; Generic Assays, Dahlewitz, Germany), IL1α-, TNFα- and non-stimulated HeLa whole cell extracts and IL6- and non-stimulated HepG2 whole cell extract were obtained by Active Motif (Carlsbad, USA). Ten microgrammes cell extract per lane were separated on a SDS-gel and transferred to a nitrocellulose membrane.

### Protein macroarray

Screening for novel autoantigens in RA was performed on hEX1 protein macroarrays derived from cDNA of human foetal brain [36] (available from engine GmbH, Hennigsdorf, Germany). Screening was carried out as described by the manufacturer. Briefly, protein macroarrays were incubated with blocking buffer (3% (w/v) milk powder in TBST (500 mM NaCl, 20 mM Tris-HCl, pH 7.5, 0.05% Tween 20)) for 1 h at room temperature followed by overnight incubation at 4 °C with patient serum (1:50 dilution in blocking buffer). Subsequently, arrays were washed three times for 20 min each, in TBST-T (TBS with 0.05% Triton X-100) and incubated 2 h at room temperature with an alkaline phosphatase (AP)-conjugated goat α-human IgG antibody (A9544, Sigma-Aldrich, St. Louis, USA) in blocking buffer for 2 h. Following three washes for 20 min in TBST-T, the arrays were incubated for 10 min in AP-buffer (1 mM MgCl_2_, 100 mM Tris-Cl, pH 9.5) and finally 5 min in 0.125 mM Attophos (Roche, Basel, Switzerland) in AP-buffer. Arrays were illuminated with an excitation wavelength of 460 nm and images were taken using a CCD camera (Fuji LAS 1000, Tokyo, Japan). Image analysis was performed with AIDA software (Raytest, Berlin, Germany). The manufacturer has defined the cutoff values according to Aida program. Each filter has an individual cutoff level defined by signals of individual dots without expression vector and dots without bacteria.

Positive positions on the arrays were scored and correlated with clone data provided by the manufacturer.

### Cloning, expression and purification of recombinant fusion proteins

Recombinant hnRNP-DL (UniProt NP_112740.1) was expressed in two bacterial clones with different variants of hnRNP-DL (amino acid 81-420 or 120-420). The hnRNP-D (AUF1) isoform p45, cloned in pTrcHis vector (Life Technologies; Carlsbad, USA), was a kind gift by Gary Brewer (School of Medicine, Wake Forest University, USA). The AUF1 p45 plasmid includes the longest transcript variant of AUF1 (UniProt NP_112738.1) and consists of 322 amino acids (45 kDa). For expression, the AUF1p45 plasmid was transformed into expression strain BL21 (DE3) pLysS cells (Merck Millipore; Darmstadt, Germany, USA) according to the manufacturer’s protocol.

The cDNA clone of hnRNP-DL was obtained from (IRAUp969E0262D, Source BioScience, Nottingham, UK). The polymerase chain reaction was performed, using the oligonucleotides primer 1: GACGACGACAAGATGGAGGTCCCGCCCAG and primer 2: GAGGAGAAGCCCGGTTAGTATGGCTGGTAATTG as primers (Biolegio, Nijmegan, Netherlands). The hnRNP-DL sequence was cloned using pET-30 Ek/LIC vector kit (Merck Millipore, Billerica, USA). Insert sequences were checked by sequencing (LGC Genomics, Berlin, Germany).

For protein expression, 1 L LB medium (containing antibiotics corresponding to the carrying expression vector) was inoculated with 5 mL overnight culture of the expression strain (*E*. *coli* LysS or -SCS1) and incubated at 37 °C with shaking until optical density of the solution was within the range of OD_600_ 0.4–0.6. Fusion protein expression was induced by adding IPTG to a final concentration of 1 mM. After 4 h, the bacterial cells were pelleted at 20.000×*g* for 10 min and stored at − 20 °C.

All proteins were expressed as His-tag fusions proteins and purified by HisPur Cobalt Resin (Thermo Fisher Scientific, Rockford, USA) in a batch process corresponding to the manufacturer’s protocol under denaturing conditions (hnRNP-DL/ AUF1).

### Enzyme-linked immunosorbent assay (ELISA)

ELISA plates (Nunc 96-well Nunc Maxisorp; Nalgene Nunc International, Rochester, NY, 1 μg protein/ well) were coated with recombinant proteins in PBS by passive adsorption at 4 °C, overnight. For sample background control plates were incubated with coating buffer only.

After washing three times with washing buffer (0.1% Tween20 in PBS, pH 7.4, 300 μL/well) and blocking with blocking buffer (5% non-fat dried milk in PBS, pH 7.4, 200 μL/well) for 90 min while shaking, the plates were then incubated with sera (1:200 dilution in 1.5% BSA in PBS, pH 7.4, 100 μL/well) for 60 min, shaking. On each plate a secondary antibody control (only serum dilution buffer) was present. Plates were washed four times with washing buffer and incubated shaking with secondary antibody (P021402-02, Dako Agilent Pathology Solutions, Santa Clara, USA, diluted 1:5000 in 5% non-fat dried milk in PBS, pH 7.4, 100 μL/well) for 45 min. Afterwards, the plates were washed five times with washing buffer. Then, they were incubated with 3.3′,5.5′-Tetramethylbenzidin (TMB, Seramun, Heidesee, Germany) for 5 min (100 μL/well) and stopped with 0.5 M H_2_SO_4_ (100 μL/well). The resulting colour reaction (from blue to yellow) was quantified with a SpectraFluor reader (Tecan; Maennedorf, Switzerland) at 450 nm and 620 nm as reference.

Each sample was quantified as mean of triplicate measurements. The difference between antigen mean value and sample background mean value, each corrected using mean value of the secondary antibody control, resulted in the net values which were used for evaluation according to the antigen-specific cutoff. The cutoff values (dotted lines) were determined by ROC analysis (GraphPad Software, version 8.0.0, San Diego, California USA, www.graphpad.com) versus other diseases (except SLE) or healthy controls with 98% specificity each. This threshold served for qualitative (positive/ negative) assessment of patient sera. These calculations were used for all ELISA analyses in human and mice.

The “Ratio mean OD positive” (Table 2) reflects the level of the positive signals in each mouse model and was calculated as the quotient of the mean value of the positive signals and the diagnostic cutoff.

To determine serum-reactivity against the citrullinated forms of the antigens by ELISA, the coated antigens were citrullinated in vitro by incubating for 3 h at 55 °C with 60 mU per well rabbit PAD (Sigma; St. Louis, USA) in 100 mM Tris, 5 mM DTT, and 10 mM CaCl_2_, while the control wells were incubated only with citrullination buffer. Afterwards, ELISA-experiments were carried out as described above

For detection of rheumatoid factor and reactivity against cyclic citrullinated peptides (α-CCP-2) commercial ELISA kits (Euroimmun, Lübeck, Germany) were applied according to the manufacturer’s protocol.

To ensure reproducibility between assays, all tests were performed with the same lot of antigen, enzyme, and TMB substrate. A cit-DL/DL-positive control serum was diluted analogously to the samples and carried with each assay. Furthermore, a secondary antibody control was included with each test, where the serum dilution buffer was incubated to allow detection of the non-specific secondary antibody background signal. The positive control and the secondary antibody control had to be within the valid range (± 10%) to assure that the measurement is correct.

### Statistical analysis

For statistical analysis Mann-Whitney test, Spearman correlation test or ROC calculation were performed (GraphPad Software, version 8.0.0, San Diego, CA, USA, www.graphpad.com).

### Immunoaffinity purification of antibodies

Autoantibodies to hnRNP-DL (amino acid 81-420) and hnRNP-D isoform p45 were affinity-purified from 10 RA patients by ELISA-elution technique. Antigen preparation was performed as described in the section “Enzyme-linked immunosorbent assay (ELISA)”. The blocked plates were incubated overnight at 4 °C with sera diluted 1:25 in PBS with 1.5% BSA, pH 7.4. Elution of antigen-bound antibodies was performed by incubation and shaking in 0.2 M Glycin-HCl pH 2.4 for 10 min. The eluate was neutralized with 1:7 elution volume 1 M Tris, pH 8.8, and immediately dialyzed against PBS, pH 7.4.

### Indirect immunofluorescence microscopy

Commercial HEp-2 cell slides (ANA HEp-2 plus; Generic Assays, Dahlewitz, Germany) were used for immunofluorescence analysis. Slides were incubated with affinity-purified α-AUF1 p45 and α-hnRNP-DL antibodies (undiluted in PBS) or rabbit α-RCK/p54 [37] antibodies (1:500; University of Florida, Gainesville, Florida, USA) overnight at 4 °C in a moist chamber. After washing, slides were incubated with α-human IgG-FITC antibody (ANA HEp-2 plus; Generic Assays, Dahlewitz, Germany) or polyclonal goat α-rabbit IgG (H+L)-Cy3 (1:50, 111-165-144, Dianova; Hamburg, Germany) antibodies.

HeLa cells were plated and exposed 1 h to 0.5 M sodium arsenite [38] and afterwards incubated with the respective primary antibodies (affinity-purified α-hnRNP-DL antibody, undiluted in PBS; rabbit α-human AUF1 peptide antibody [19], 1:1000; mouse α-ATXN2 antibody [39], 1:200; mouse α-hnRNP-A2/B1, 1:500; Acris Antibodies, San Diego, USA). For immunofluorescence analysis, corresponding FITC- and Cy3-coupled, secondary antibodies were used as previously described [40].

Preparations were analysed at 400-fold magnification with a LSM510 fluorescence microscope (Carl Zeiss; Jena, Germany) fitted with the appropriate filter sets for FITC and Cy3.

### Immunohistochemical analysis

We analysed an α-hnRNP-DL antibody (ARP4085_T100; Aviva Systems Biology; San Diego, USA) in 1:50 dilution on a tissue microarray (TMA, Provitro, Berlin, Germany) with paraffin sections of human synovial tissue from patients with rheumatoid arthritis (*n* = 10), osteoarthritis (*n* = 12) and from healthy donors (*n* = 4). Immunostaining of the antibody was performed according to the manufacturer’s protocol. For visualization, the Novolink™ Polymer Detection System (RE-7140-CE; Leica Biosystems, Nussloch, Germany) was used. Slides were analysed with a CX41 microscope (Olympus, Tokyo, Japan).

### Preparation of synovial tissue

Synovial tissue was collected from a patient with rheumatoid arthritis (joint biopsies, Department of Rheumatology and Clinical Immunology, Charité, Berlin). Lysis of synovial tissue was performed in M-PER Mammalian Protein Extraction Reagent. (78501; Thermo Fisher Scientific Inc., Rockford, IL, USA) with protease inhibitor cocktail (P8340; Sigma, St. Luis, USA), 100 μM Na3VO4, 150 mM NaCl, and 1 mM DTT, followed by mechanical homogenization with an Ultra Turrax (T25; IKA, Staufen, Germany) three times for 1 min. After centrifugation (1 min, 17,000×*g*) the supernatant was collected. To enhance solubility of proteins, the pellet was homogenized three times for 1 min with ultrasound (VibraCell; Sonics and Materials, Danbury, USA) in 8 M urea. Ten microgrammes of each, the supernatant and the pellet homogenate, were separated together in one lane on a SDS-gel and transferred on a nitrocellulose membrane.

### Gel electrophoresis, immunoblotting and immune detection

Total protein was measured using the Bradford assay (Roth, Karlsruhe, Germany). Equal amounts of protein in sample buffer [41] were separated on 12.5% SDS minigels with 4% stacking gel in SDS running buffer (25 mM Tris, 0.2 M Glycine, 0.1% (w/v) SDS) for 40–50 min at 25 mA in a gel apparatus (Mighty Small II, Amersham Pharmacia, Uppsala, Sweden). Afterwards, proteins were transferred to nitrocellulose membranes (BA85; Schleicher & Schuell, Dassel, Germany) using a tank blot system (TE22; Hoefer, Holliston, USA) with tankblot buffer (15 mM Tris, 0.1 mM glycine) for 1 h and 400 mA with stirring and water cooling. After protein transfer, the Western blot was blocked for 1 h in blocking solution (3% w/v non-fat dried milk in PBS, pH 7.4) and incubated with an α-hnRNP-DL antibody (ARP40586_P050; 1:500; Aviva Systems Biology, San Diego, USA), hnRNP-DL-specific rabbit antibody serum (peptide motif MEDMNEYSNIEEFAEGSK, contained in all hnRNP-DL isoforms, 1:100, Thermo Fisher Scientific, Rockford, USA) or an α-deiminated arginine antibody (ABAP Kit; 1:500; Modiquest, Oss, Netherlands) diluted in blocking solution overnight at 4 °C. After washing three times for 10 min with washing buffer (PBS, pH 7.4, 0.05% Triton X-100) the blot was incubated for 1 h with secondary antibody (rabbit or mouse horse radish peroxidase conjugate, Dako Agilent Pathology Solutions, Santa Clara, USA) 1:1000 diluted in 3% w/v non-fat dried milk in washing buffer. After washing five times for 5 min with washing buffer, chemiluminescence detection was performed with Roti Lumin substrate (Roth, Karlsruhe, Germany) according to the manufacturer’s instructions.

### Global sequence alignment

With the help of the program “Needle”, version 2019 [42], which can be accessed online via the EMBOSS website [43] (http://www.ebi.ac.uk/Tools/psa/emboss_needle/; retrieved on 04 April 2020), we performed a global sequence alignment by Needleman-Wunsch algorithm [44]. The amino acid sequences of the two hnRNP proteins hnRNP-D (isoform A, 355 AA) and hnRNP-DL (isoform 1, 420 AA) were analysed for homologous sequence regions.

## Results

### Protein macroarray screening identifies the hnRNP-DL protein as a novel autoantigen targeted in rheumatoid arthritis (RA)

Sera from 26 RA patients and 40 control subjects, including osteoarthritis (OA) patients (*n* = 20) and self-reported healthy blood donors (*n* = 20), were analysed on protein macroarrays [36]. The 20 most sensitive autoantigens only found in the RA group are listed in the Additional file 2.

We identified α-hnRNP-DL with second highest intensity score. HnRNP-A2/B1 and hnRNP-D (AUF1) have already been described as autoantibody targets in RA [11, 19]. Structure of hnRNP-DL and sequence alignment with hnRNP-D is shown in Additional file 1: supplementary Figure 1. One of two different hnRNP-DL clones, expressing the protein fragment from amino acid 81 to 420, revealed autoantibody reactivity in 20% of RA sera (Additional file 2). This hnRNP-DL fragment was termed hnRNP-DL_mir_ (major immunogenic region). Isoform hnRNP-DL2 (amino acid 120-420) could not be detected by RA sera.

### Autoantibodies against native and citrullinated hnRNP-DL are predominantly present in sera of systemic lupus erythematosus (SLE) and RA patients

**Fig. 1:**
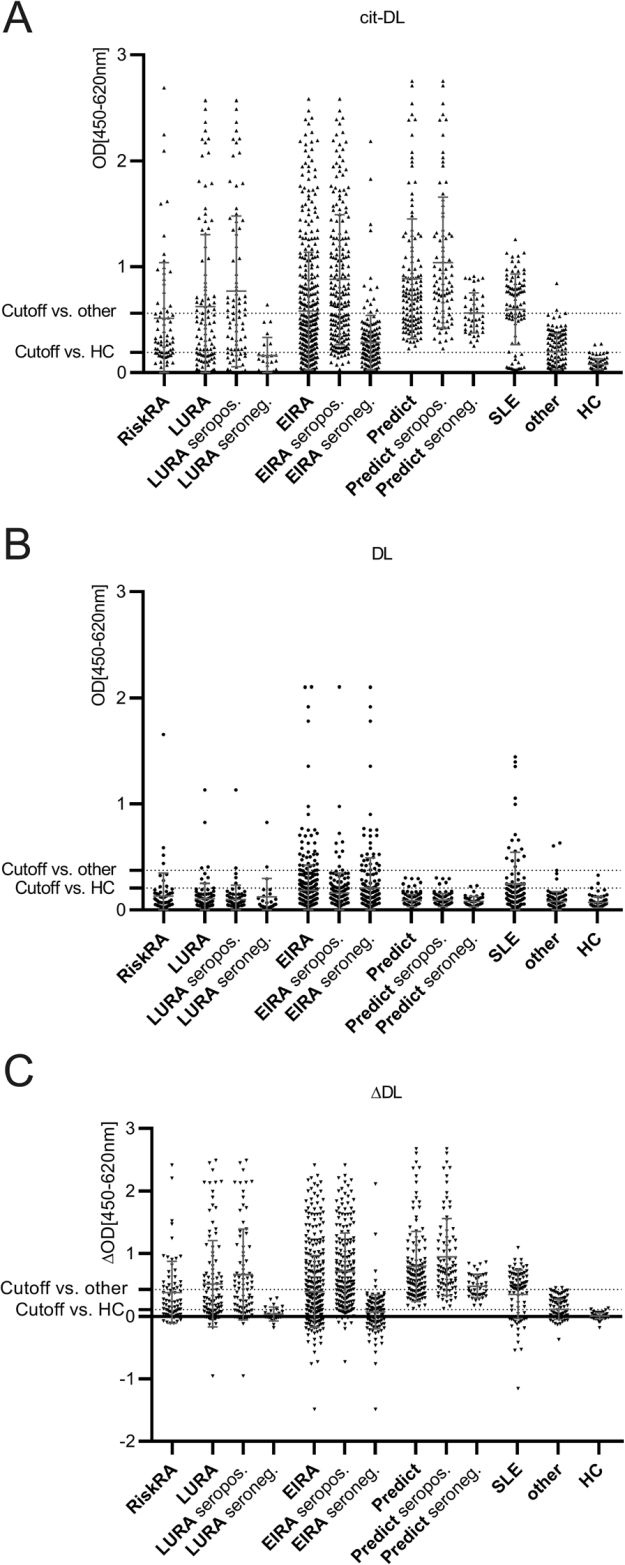
Distribution of ELISA signals of α-hnRNP-DL_mir_ autoantibodies. Reactivities were predominantly found in SLE and RA. Prevalence of citrullinated α-hnRNP-DL_mir_ (cit-DL) (**A**), α-hnRNP-DL_mir_ (DL) (**B**) and the difference between cit-DL and DL signal (ΔDL) (**C**) in sera from Risk-RA patients (*n* = 71), early RA patients (LURA *n* = 106; EIRA *n* = 404), established RA patients (predict *n* = 127), SLE patients (*n* = 89), other diseases (*n* = 127) and healthy controls (*n* = 86) determined by ELISA. The dotted lines mark the cutoff versus other diseases (except SLE) or healthy controls with 98% specificity each. OD, optical density; SLE, systemic lupus erythematosus

To verify the results from protein macroarray screening, hnRNP-DL_mir_ was expressed in *E*. *coli* BL21(DE3)pLysS, purified and tested for reactivity in ELISA as native (DL) and citrullinated protein version (cit-DL), using 1010 sera obtained from Risk-RA cohort (*n* = 71), from early RA cohorts (LURA *n* = 106; EIRA *n* = 404), from an established RA cohort (predict *n* = 127), control cohorts of other autoimmune diseases (*n* = 216) and from healthy controls (*n* = 86). Since citrullinated antigens, among them hnRNP-A2/B1 [18], are the most specific targets in RA, we analysed autoantibody responses against cit-DL, with the highest signalling and positivity found in the early and established RA cohorts (64–100%). With special focus on the seropositive and seronegative RA patients only α-cit-DL signals differ significantly within all investigated RA cohorts, not α-DL values (Fig. 1A/B; Additional file 1: supplementary Table 1). Although α-cit-DL signals of seronegative patients were lower than those of seropositive patients, they were still significantly higher than in other diseases in EIRA and predict cohort (Additional file 1: supplementary Table 2).

The majority of α-DL was found in sera of patients with SLE (34%) and RA (6–21%) and in patients with psoriasis arthritis (15%), patients with MS (5%) and scleroderma (5%) as well as healthy controls (2%) (Fig. 1B; Additional file 1: supplementary Figure 2). Interestingly, we obtained very different sensitivities within the four investigated RA cohorts whereby Risk-RA- (13%) and EIRA cohort (21%) showed the highest sensitivities. Cohorts under certain therapy or advanced disease duration showed lower values (LURA 8%/predict 6%).

Noticeable 58% of the SLE patients, using the cutoff level versus other diseases, were α-cit-DL positive (α-DL 18%), although 98% of the tested SLE sera were α-CCP-2-negative. We determined the difference between the ELISA signals, to get a value that describes the relationship between α-cit-DL and α-DL. This value we named CN_DL_-Index (ΔDL), shown in Fig. 1C, with the highest values detected in the RA cohorts. In established RA (predict), the highest CN_DL_-Index (sensitivity, 100%/72% versus healthy controls/other diseases) and exclusively positive values were detected. In contrast, 11–20% of early RA patients (Risk-RA/EIRA/ LURA) had a negative CN_DL_-index, where α-DL was higher than α-cit-DL. Besides, only SLE patients and single exceptions in other diseases had a negative CN_DL_-index below − 0.1. In the early RA cohort EIRA, the CN_DL_-Index correlated positively to α-cit-DL and there, exclusively in the seronegative EIRA negatively to α-DL response (Additional file 1: supplementary Figure 3).

### Anti-cit-DL and CN_DL_-Index correlated with parenchymal changes in lung/shared epitope and identified people at risk to develop RA

Anti-DL autoantibodies were detectable in early RA. Therefore, we investigated α-CCP2-positive healthy subjects with musculoskeletal symptoms, classified as Risk-RA cohort, differentiating between subjects developing arthritis during follow-up and those remaining healthy without arthritis diagnosis. Further, we analysed α-DL autoantibody association with certain risk factors for RA. We plotted respectively α-cit-DL, α-DL and the CN_DL_-index in the LURA cohort with the parenchymal changes in the lung and in the EIRA cohort with the genetic risk factor shared epitope.

**Fig. 2:**
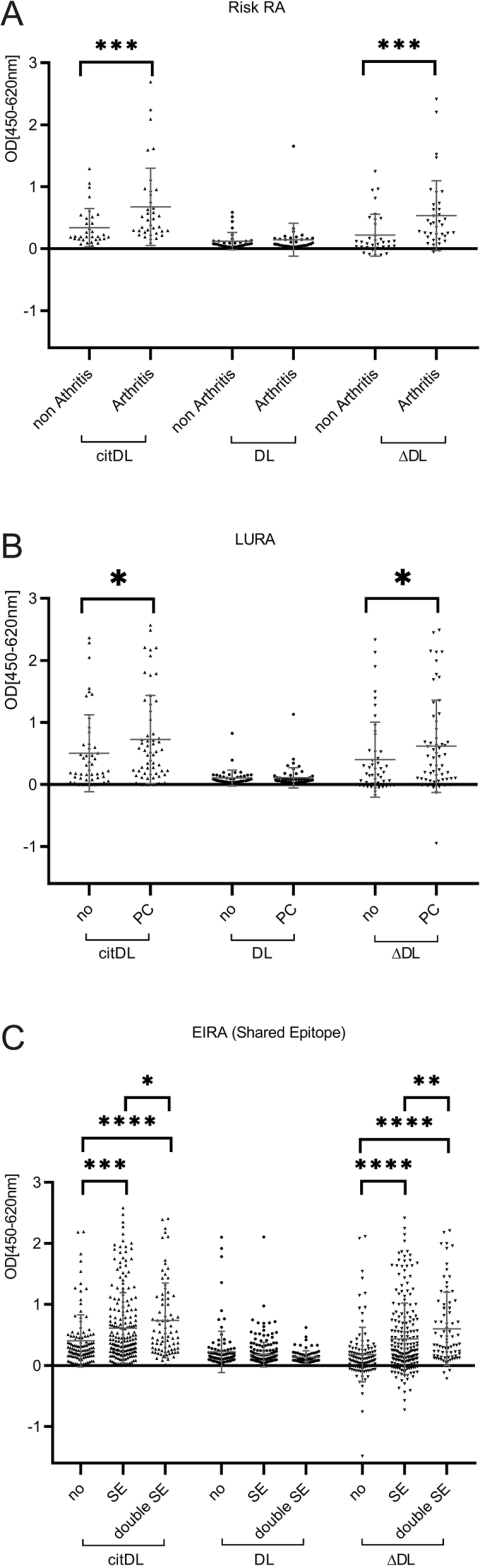
Anti-citrullinated hnRNP-DL_mir_ autoantibodies are detectable even before the onset and in early status of disease. **A**–**C** Anti-citrullinated hnRNP-DL_mir_ (cit-DL), α-hnRNP-DL_mir_ (DL) and ∆ OD between cit-DL and DL (ΔDL) were measured by ELISA. **A** In Risk-patients of arthritis the OD levels of cit-DL and ΔDL before onset are significantly specific in the patient group where the arthritis has already been diagnosed compared to the group without diagnosis (*n* = 71; non-arthritis *n* = 34/arthritis *n* = 37; Mann-Whitney *U*; cit-DL median_non Arthritis_ = 0.19/median_Arthritis_ = 0.46; *p* = 0.0006; NC-index median_non Arthritis_ = 0.10/median_Arthritis_ = 0.38; *p* = 0.0003). **B**, **C** Cit-DL and ΔDL are significantly associated with parenchymal changes in the lung of early RA patients of the LURA cohort (**B**; *n* = 106; no *n* = 48/PC *n* = 58; Mann-Whitney *U*; cit-DL median_no_ = 0.23/median_PC_ = 0.53; *p* = 0.0340; NC-index median_no_ = 0.16/median_PC_ = 0.44; *p* = 0.0332) and with and shared epitopes of the early RA patients of the EIRA cohort (**C**; *n* = 404; no *n* = 112/SE *n* = 213/double SE *n* = 79; Mann-Whitney *U*; cit-DL median_no_ = 0.27/median_SE_ = 0.36; *p* = 0.0003, median_no_ = 0.27/median_double SE_ = 0.54; *p* < 0.0001, median_SE_ = 0.36/median_double SE_ = 0.54; *p* = 0.0453; NC-index median_no_ = 0.11/median_SE_ = 0.21; *p* < 0.0001, median_no_ = 0.11/median_double SE_ = 0.34; *p* < 0.0001, median_SE_ = 0.21/median_double SE_ = 0.34; *p* = 0.0061). Mann-Whitney *U* test was performed for analysing significance of indicated groups (*p < 0.05, **p < 0.01, ***p < 0.001 ****p < 0.0001). OD, optical density; ns, not significant; PC, parenchymal changes in lung; SE, shared epitope

In the Risk-RA cohort, α-cit-DL and CN_DL_-Index were significantly elevated in progressors (Fig. 2A), in the LURA cohort in patients with parenchymal lung changes (Fig. 2B) and in the EIRA in patients with shared epitope, particularly in those carrying two copies (Fig. 2C). No significant differences were found for α-DL antibodies.

### High α-DL autoantibody levels found in 6-month EULAR responders for methotrexate (MTX) or Enbrel® treatment

**Fig. 3:**
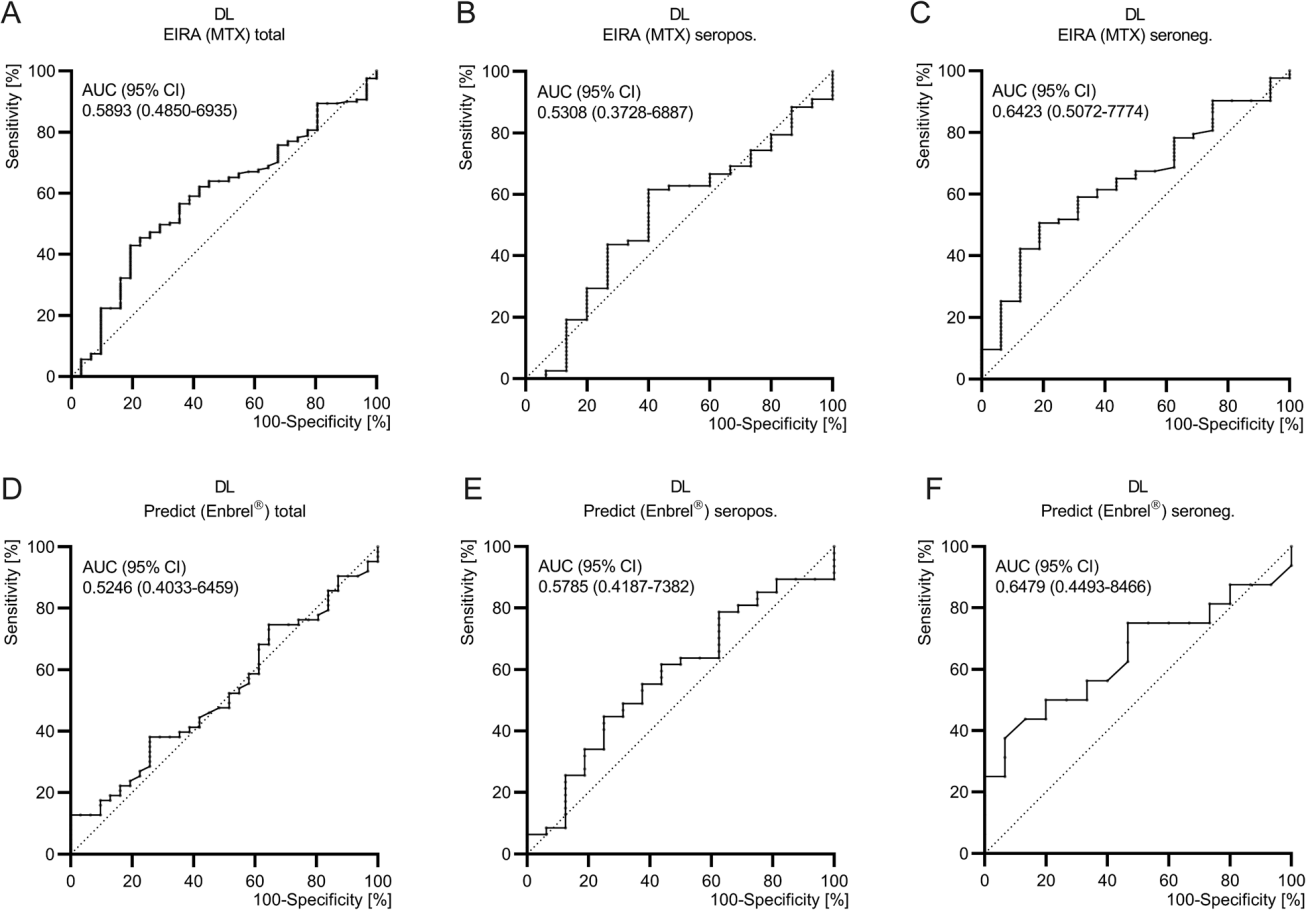
Diagnostic performance of α-hnRNP-DL_mir_ (DL) for the detection of therapy response. High baseline titre against α-hnRNP-DL_mir_ (DL) is rather present in 6-month EULAR Responder RA patients who had received MTX or α-TNF inhibitor therapy (Enbrel®). **A**–**C** α-DL were measured by ELISA in patient sera from the EIRA cohort treated with MTX (*n* = 192) with 161 EULAR responder and 31 EULAR non-responder among 6 months. Above these values, ROC analyses were performed for detecting DAS28 therapy response. **D**–**F** α-DL were measured by ELISA in patient sera from the predict cohort treated with Enbrel® therapy with 6-month EULAR response data (*n* = 94, responder *n* = 63, non-responder *n* = 31). Based on the signals, ROC analysis was performed for detecting DAS28 therapy response. OD, optical density; vs., versus; RA, rheumatoid arthritis; MTX, methotrexate; seropos., rheumatoid factor IgM and/or α-CCP-2 positive patients; seroneg., rheumatoid factor IgM and α-CCP-2 negative patients

We examined our biomarkers (α-cit-DL, α-DL and CN_DL_-index) with therapy data of the EIRA and predict cohort. One hundred and ninety-two MTX-treated EIRA patients were analysed (Fig. 3A–C; Additional file 1: supplementary Figure 4A/B). The ROC analysis of α-DL signals reached 12% sensitivity with 90% specificity, using the RA-specific cutoff level (OD 0.371) for detecting MTX response. ROC results got more significance for detecting MTX responses in the seronegative group (cutoff 0.371; 16% sensitivity, 94% specificity; Additional file 1: supplementary Table 6).

Because α-DL correlated negatively to the CN_DL_-Index in the seronegative group (Additional file 1: supplementary Figure 3), we analysed MTX-treated EIRA patients with negative CN_DL_-index. Eighty-seven percent of these patients were responders. We reached sensitivities in a range of 15–33% (100/75% specificity) to detect MTX response (Additional file 1: supplementary Table 7).

In the predict cohort (Enbrel®-treatment) no CN_DL_-index/response association were found since all patients had equally high positive CN_DL_-index and none of them negative values. ROC analysis of α-cit-DL or CN_DL_-index showed no specific response cutoff. But with α-DL, we identified 23% of the EIRA patients as MTX responder and in the seronegative group 25% (90% specificity). Among the established RA cohort (predict), α-DL reached 13% sensitivity and even 25% within the seronegative group for the detection of Enbrel® response (100% specificity; Fig. 3D–F; Additional file 1: supplementary Table 8).

### Anti-cit-DL and α-DL increase the serodiagnostic sensitivity in early RA

All RA cohorts were analysed to determine diagnostic sensitivities of α-cit-DL and α-DL, in RF IgM/α-CCP-2-seropositive and -negative patients.

The calculated cutoff level versus healthy controls (96% specificity), identified 80% of the subjects in the Risk-RA cohort, which are exclusively α-CCP-2-positive. In the LURA/EIRA cohort 32/73% of the seronegative patients were identified. In the predict cohort, all patients could be identified with one of our biomarkers and α-DL response was on average the lowest (6%). In SLE patients, 84% in total were detected (α-DL, 34%; α-cit-DL, 80%). In other autoimmune diseases, about half (48%) of the patients were detected in total with our biomarker set.

Using the cutoff level versus other diseases (96% specificity), we detected 51% of the seronegative established RA patients and 8–17% of the early RA patients (Table 1).

**Table 1:** Sensitivity of α-citrullinated hnRNP-DL_mir_ (cit-DL), α-hnRNP-DL_mir_ (DL) autoantibodies and Δ OD between cit-DL and DL (ΔDL) in sera from Risk-RA patients, early RA patients (LURA/ EIRA), established RA patients (predict), SLE patients (*n* = 89), other diseases and healthy controls determined by ELISA. Sensitivities are expressed as percentages, with a 98% specificity, and were calculated using two cutoffs in each case, first, against healthy controls (to the left of the slash) and second, against other diseases except SLE (to the right of the slash). Total DL is the combined antibody reactivity and describes the proportion of patients that one detects positive overall with the combination of all three biomarkers α-cit-DL, α-DL and/or ΔDL

	cit-DL	DL	ΔDL	total DL	RF and/or CCP positive				RF and CCP negative			
					cit-DL	DL	ΔDL	total DL	cit-DL	DL	ΔDL	total DL
**Risk-RA**	*n* = 71				*n* = 71				*n* = 0			
**% pos.**	70/28	13/6	68/34	80/39	70/28	13/6	68/34	80/39	-	-	-	-
**LURA**	*n* = 106				*n* = 81				*n* = 25			
**% pos.**	64/38	8/4	64/40	69/42	77/48	7/2	77/52	80/53	24/4	12/8	24/0	**32/8**
**EIRA**	*n* = 404				*n* = 202				*n* = 202			
**% pos.**	80/33	21/9	68/31	84/40	94/59	17/5	89/59	96/63	65/7	25/14	46/2	**73/17**
**Predict**	*n* = 127				*n* = 86				*n* = 41			
**% pos.**	100/67	6/0	100/72	100/72	100/77	6/0	100/83	100/83	100/46	5/0	100/51	**100/51**
**SLE**	*n* = 89											
**% pos.**	80/58	34/18	72/57	84/73								
**other**	*n* = 127											
**% pos.**	45/2	4/2	41/2	48/4								
**HC**	*n* = 86											
**% pos.**	2/0	2/0	2/0	5/0								

*HC* healthy controls, *RA* rheumatoid arthritis, *SLE* systemic lupus erythematosus

### Localization and expression of hnRNP-DL in different cell lines and synovial tissue

**Fig. 4:**
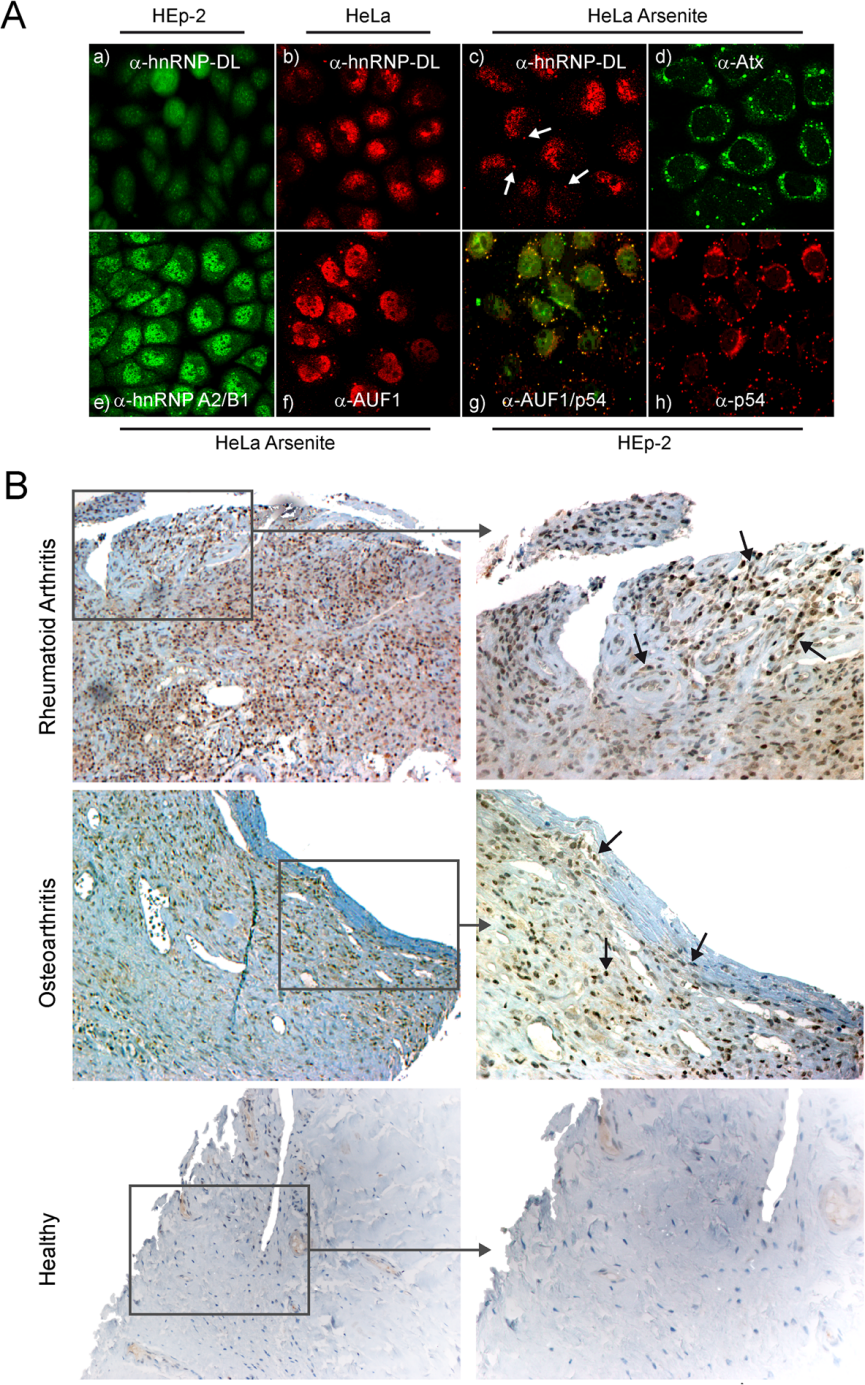
Localisation and expression of cytokine-regulated, stress granule protein hnRNP-DL_mir_ in cells, and synovial tissue. Anti-human hnRNP-DL_mir_ antibodies detect stress granules in immunofluorescence microscopy. Staining with an affinity-purified α-human hnRNP-DL_mir_ antibody was performed in HEp-2 (a) and HeLa cells (b). HeLa cells were treated with 0.5 mM sodium arsenite to induce stress granules and stained with affinity-purified α-human hnRNP-DL_mir_ antibodies (c), mouse α-human ATXN2 antibodies 63 (d), mouse α-human hnRNP-A2/B1 antibodies (e) and α-human AUF1 peptide-specific rabbit serum 19 (f). Co-localization of AUF1 and stress granules/P-bodies. Staining of HEp-2 cells with α-RCK/p54 64 antibodies (g) and double staining of HEp-2 cells with affinity-purified α-human AUF1 (green) and α-RCK/p54 64 antibodies (red) (h). Merged sections are visible in yellow. **B** Expression of hnRNP-DL in synovial tissue from a patient with rheumatoid arthritis, a patient with osteoarthritis and a healthy subject, each in 20-fold and detail in 40-fold magnification

Affinity-purified α-DL autoantibodies from RA patient sera were used for localization of hnRNP-DL in HeLa- and HEp-2 cells. Sparing the nucleoli in interphase cells, staining with the α-DL autoantibodies showed a nucleoplasmic staining with large speckles (Fig. 4A; a, b). However, the nucleoplasmic staining produced by α-hnRNP-D (α-AUF-1) and α-hnRNP-A2/B1 antibodies was more homogeneous (Fig. 4A; e, f) and stained as well as α-DL autoantibodies discrete cytoplasmic foci when cells were stressed by arsenite (Fig. 4A; c, e, f). Notably, the co-localization experiment showed α-DL antibodies stained a subset of cytoplasmic stress granules (Fig. 4A; c), independent of size and localization. HnRNP-D could be detected in nearly all granules (Fig. 4A; g, yellow), like the controls Ataxin2 and RCK/p54 (Fig. 4A; d/h).

Since previous studies demonstrated hnRNP-A2/B1 and hnRNP-D to be highly expressed in synovial tissue of RA patients and arthritic mice [19, 28, 45, 46], we analysed the expression of hnRNP-DL in the human joint. Specific rabbit antibodies recognizing hnRNP-DL 1 and 2 expression were tested by immunohistochemistry in synovial tissue of RA and OA patients and from healthy controls (Fig. 4B). These analyses revealed hnRNP-DL to be highly expressed in RA tissue. Nuclear and cytoplasmic expression was seen in cells of RA synovial tissue, in contrast to the exclusive nuclear staining observed in OA and normal tissue (Fig. 4B, arrows).

We further investigated the expression of hnRNP-DL under inflammatory conditions in IL1α- and TNFα-stimulated HepG2-, as well as in IL6-stimulated HeLa cells by immunoblotting (supplementary Fig. 5 A/B). TNFα and particularly IL1α upregulate, whereas IL6 downregulates the expression of hnRNP-DL and furthermore induces its degradation.

We further detected citrullinated proteins of the same molecular weight as of hnRNP-DL (supplementary Fig. 5 B) in the synovial tissue. The molecular weights of the detected DL bands in supplementary Figure 5 B do not correspond to the isoforms in supplementary Figure 5A; they may be other DL isoforms that have not been studied in detail.

### Anti-DL in animal models of RA and SLE with association to TLR7/9 and MyD88 — supports reference to clinical pain

Anti-cit-DL/α-DL autoantibodies, in baseline samples, are associated with pain VAS after 6 months of various treatments of EIRA patients (Additional file 1: supplementary Table 3-5).

Therefore, we wanted to study the production of α-DL autoantibodies in the context of TLR and MyD88-knock-out mice, known to be involved in pain pathway [47, 48]. Because hnRNP-DL is highly conserved in human and mouse (similarity 98.5% [44]), we analysed α-DL in sera of mouse models of RA and SLE (Table 2).

**Table 2:** Frequency of autoantibodies against recombinant hnRNP-DL_mir_ in sera from different RA and SLE mouse models

Mouse model	Model	Autoantigen(s) assayed	No. of sera tested	% Positive	Ratio, Mean OD positive^b^
SKG (-/+ Zymosan)	RA	hnRNP-DL_mir_	8/8	25/50	2.48/1.23
Balb/c (IL-1Ra^−/−^)	RA	hnRNP-DL_mir_	36	100	7.89
MRL-lpr	SLE	hnRNP-DL_mir_^a^	20	85	4.22
MRL-lpr (MyD88^−/−^)	SLE	hnRNP-DL_mir_^a^	20	10	1.3
MRL-lpr (TLR9^−/−^)	SLE	hnRNP-DL_mir_	4	50	2.42
MRL-lpr (TLR7^−/−^)	SLE	hnRNP-DL_mir_	7	43	2.55
MRL-lpr (TLR7/9^−/−^)	SLE	hnRNP-DL_mir_	7	0	-
C57BL/6 lpr	SLE	hnRNP-DL_mir_^a^	12	33	2.46
C57BL/6 lpr (SIGIRR/TIR8^−/−^)	SLE	hnRNP-DL_mir_^a^	12	83	6.83
C57BL/6 (-/+ R848)	TLR7/8 agonist	hnRNP-DL_mir_	10/10	0/10	-/1.39

*RA* rheumatoid arthritis, *SLE* systemic lupus erythematosus, *SIGIRR/TIR8* Single Ig IL-1-related receptor/Toll/interleukin-1 receptor 8, *R848* SIGIRR TLR7/8 agonist, *MyD88* myeloid differentiation primary response gene 88, *TLR* Toll-like receptor

^a^Additionally citrullinated hnRNP-DL_mir_ were tested. In no case, citrullination of hnRNP-DL_mir_ resulted in a higher signal compared to the native hnRNP-DL_mir_ form (no additional reactivity)

^b^Ratio mean OD positive reflects the level of the positive signals in each mouse model and was calculated as the quotient of the mean value of the positive signals and the diagnostic cutoff

In zymosan-treated SKG-mice [49], α-DL autoantibodies were twice as frequent (50%) compared to the less severe arthritis model without zymosan induction (25%).

Interestingly, in the interleukin-1 receptor antagonist-deficient (IL-1Ra^−/−^)-mouse arthritis model we found high signals of α-DL autoantibodies in all mice tested.

MRL/lpr-mice produce antibodies against hnRNPs [50] and snRNPs [51] α-DL autoantibodies were detectable in 85%, while none of them were positive for the citrullinated protein version.

We analysed sera from TLR7-, TLR9-, and TLR7/TLR9-double deficient lupus-prone MRL/lpr-mice. This investigation revealed that α-DL autoantibodies were TLR7/-9 dependent and only completely absent in the double deficient mice, while they remained detectable in about 50% of the single TLR7- or TLR9-knock-out MRL/lpr-mice. MyD88 plays a central role in TLR-pathway [52]. We tested MyD88-deficient mice, which did not produce α-DL autoantibodies except two mice with very low titre. Further, we tested knock-out mice of Toll interleukin-1 receptor 8 (TIR8, SIGIRR, IL1R8), a negative regulator of TLR-IL1-receptor family signalling. Genetic inactivation of this protein, which is associated with severe autoimmunity and high autoantibody production [53], increased prevalence of α-DL autoantibodies by 50%, with a three times higher mean level of ELISA signal intensity (Table 2).

## Discussion

RA antibody systems are remarkably diverse, characterized by the presence of those against native proteins as well as those containing posttranslational modifications (PTMs) [54, 55]. While current models of RA have embraced PTMs as core principles of pathogenesis [54, 56], α-native protein antibodies are not adequately explained by the PTM-centric paradigm of autoantigen selection. The direct α-citrullinated protein-antibody response may depend on the presence of permissive factors, i.e., a genetic predisposition, as has been shown for α-cit-DL with its shared epitope (SE)-dependency and the continued production of modified antigen. Chronic bacterial infection, such caused by *Aggregatibacter actinomycetemcomitans* [57] or *Porphyromonas gingivalis*, which can citrullinate hnRNPs [58] or smoking [59, 60], leading to overexpression of hnRNPs, as shown by our results with overexpression and citrullination of hnRNP-DL in RA joint.

In early RA, a serodiagnostic gap of 50–60% [61–64] left by using RF IgM/ α-CCP-2 assays. This is of particular importance as patients considered to be autoantibody negative may erroneously not be diagnosed as having RA due to inappropriate therapeutic measures. In recent years, novel biomarkers have been described with sensitivities between 16 and 67% in α-CCP-2-negative RA cohorts [65]. However, the clinical utility of these biomarkers is questionable because diagnostic specificities are largely unknown and will have to be shown in further studies. RF IgM/ α-CCP-2-seronegative RA patients became seropositive by a combination of our biomarker set (α-cit-DL, α-DL, CN_DL_-index). In the clinical autoantibody testing, the new biomarker can be used for detecting people “at risk” for RA, and for early and established RA, reducing the sensitivity gap of RF IgM/ α-CCP-2-seronegative patients (sensitivity RF IgM/α-CCP-2 negative LURA/EIRA/predict 32%/73%/100%; Table 1).

It has already been published that it is important to study the citrullinated signal adjusted from the unmodified protein/peptide signal, to obtain the specific signal, which is added or reduced by the modification. It has been shown that these autoantibodies occur specifically in RA, but without clinical associations such as therapy response [30, 58].

Therefore, we have introduced and tested a new biomarker CN_DL_-index which measures the difference of α-cit-DL and α-DL ELISA OD levels, covering both antibodies against citrullinated epitopes and structural citrullinated epitopes (SCEs). Negative CN_DL_-index was detectable at an early timepoint of arthritis and even before arthritis starts. Moreover, RA patients with such negative CN_DL_-index tended to respond positively to MTX/Enbrel® therapy. As RA progresses, the CN_DL_-index became increasingly positive and was associated with SE, parenchymal changes in lung and lower the response to MTX therapy.

Citrullination is a hydrolytic reaction, the target protein mobility in SDS-PAGE will shift, yielding a noncharged citrulline amino acid and neutral urea through the hydrolysis of the strongly basic positively charged side chain of arginine by water. This charge shift affects protein structure, protein-protein interactions, and hydrogen bond formation, and it may cause protein denaturation [66, 67]. This study suggests an alternative model to the PTM-centric model in which the antigen is initially targeted independent of citrullin itself, but may be depend on a structural change induced by cryptic PTM that causes the autoantibody binding. Demonstrably, the sensitivity of α-cit-DL within the tested SLE patients was 58%, almost three times higher than the sensitivity of α-DL (18%), calculated with the cutoff versus other diseases, although 98% of the tested SLE sera were α-CCP-2-negative. Citrullination leads to formation of a new SCE, whose recognition is independent of directly targeting the citrulline site. This new form of α-SCE autoantibodies may explain the shift from an initial native autoantibody response against PTMs. DNA, RNA and TLR7/9 activation are required to generate α-hnRNP-specific B cells and this complex induced RA in a pristane-induced arthritis model of RA [68]. Interestingly, MyD88 deficiency leads to reduction of pain [47], which may explain the correlation of α-DL with pain VAS after 6 months in the EIRA cohort. Autoantibodies against DL did not correlate to RF IgM or α-CCP-2 or SE. These antibodies can be used specifically in the seronegative group to predict the therapeutic outcome and pain level after 6 months of treatment.

The α-DL autoantibody level disappeared in the course of RA, inversely the α-cit-DL autoantibody level increased, independently from the therapeutic regime. Therefore, future therapies utilizing tolerance induction may use native RA autoantigens in “high risk” individuals. Epitope spreading to PTM autoantigens can be blocked in the major mouse models of SLE and RA that we have tested, and this could be analysed experimentally with hnRNP-DL in future studies. Native antigens as part of stress granules are used in existing models of experimental arthritis to induce arthritis, but not the citrullinated antigens [68]. SE and specific exogenous factors are missing in the studied animal models of RA and SLE, explaining the lack of ACPAs and SCE autoantibodies. Anti-native protein antibodies may represent markers for the detection of risk people in the earliest pre disease of RA, preceding the development of the ACPA response, predicting a mild disease. For α-hnRNP-A2/B1 autoantibodies, an association to less erosive disease, exclusively in early RA, has already been published [18, 29]. Recently, several more reactivities against native proteins in RA have been published [55]. Therefore, it is important to measure other hnRNP autoantibodies and in combination in future studies to evaluate them for personalized medicine.

## Conclusions

These new data suggest that hnRNP-DL is a novel TLR7/9-dependent autoantigen found predominantly in RA and SLE and in mouse models of inflammatory rheumatic diseases. Our studies on hnRNP-DL have shown that citrullination can lead to structural epitopes (SCE) that can be recognized by α-CCP-2-negative SLE patients. By using the combined assay consisting of citrullinated hnRNP-DL and native hnRNP-DL, we increase the serodiagnostic sensitivity in RA patients who are negative for RF and α-CCP-2 autoantibodies. We demonstrated that autoantibodies against hnRNP-DL have prognostic value for the differential diagnosis of RA, especially in early disease. Immunofluorescence analyses revealed that hnRNP-DL is part of stress granules that can trigger inflammatory processes in RA. Our results indicate that truncated, possibly citrullinated, immunogenic hnRNP-DL can be detected in synovial tissue.

We hypothesize that hnRNP autoantibodies generated by patients with systemic autoimmune diseases are directed against mRNA decay complexes that are part of the stress granules. We hypothesize that increased formation and structural modification of such protein complexes by bacterial or human enzymes (e.g., in inflammatory processes with overexpression of IL1α and/or TNFα) may lead to a pathogenic autoimmune response against a structurally altered form of native hnRNP-DL and that SCE epitopes may arise before the temporal increase in PTM-specific targets. In conclusion, the introduction of a CN-index biomarker that measures specific anti-citrulline signalling in autoantigens will help to objectively facilitate early RA treatment decisions that are not measurable with current commercial ACPA assays.

Further autoantibody studies with additional hnRNP family proteins in native and citrullinated form should follow to identify new subsets of reactivities in RA patients. In addition, the clinical significance of the structural epitopes should be investigated in detail.

## Supplementary Information

**Additional file 1: Figure 1.** Sequence, structure and major immunogenic region (mir) of hnRNP-D and hnRNP-DL. A, Schematic representation of hnRNP-D (isoform p45), hnRNP-DL and the different recombinant hnRNP-DL variants studied. The main structural features are highlighted. Mir-region is the major immunogenic region, RBD1 and RBD2 are RNA-binding domains 1 and 2, Gly-rich is the C-terminal glycine-rich region of the proteins. B, Global amino acid sequence alignment of hnRNP-D and hnRNP-DL1 (isoform 1). HnRNP-D and -DL share 89.1% similarity by sequenc e[1]. Regions “mir”, “RBD1”, “RBD2” and “Gly-rich” are highlighted. **Figure 2.** Characterisation of autoantibodies against, A, citrullinated α-hnRNP-DL_mir_ (cit-DL), B, α-hnRNP-DL_mir_ (DL) and C, ∆OD between cit-DL and DL (ΔDL) determed by ELISA in sera of other diseases (n=127; MS n=20, reA n=7, Sclero n=20, Sjö n=20, PsA n=20, MB n=20, OA n=20). The dotted lines markes the cutoff vs. other diseases (except systemic lupus erythematosus) or healthy controls with 98% specificity each. OD, optical density; nm, nano meter; vs., versus; MS, multiple sclerosis; reA, reactive arthritis; Sclero, scleroderma; Sjö, Sjögren´s syndrome; PsA, psoriasis arthritis; MB, ankylosing spondylitis; OA. Osteoarthritis. **Table 1**. Mann Whitney U-test of (cit) α-hnRNP-DL_mir_-OD signals of seropositive and seronegative data sets of RA-cohorts. **Table 2.** Mann Whitney U-test of cit α-hnRNP-DL_mir_-OD signals of seronegative data sets of RA-cohorts and data sets of other inflammatory diseases. **Figure 3.** XY-Plot and Spearman Correlation of citrullinated or native α-hnRNP-DL_mir_ versus ΔhnRNP-DL_mir_ for the early RA cohort EIRA (A/D; n=404), the seropositive EIRA sera (B/E; n=202) and the seronegative EIRA sera (C/F; n=202). **Table 3.** Spearman correlation of the early RA sera of the EIRA cohort (n=404). The results are given as R value (left of slash) with the corresponding p-value (right of slash). **Table 4.** Spearman correlation of the 242 EIRA sera treated with MTX (α-CCP2 positive n=133, α-CCP2 negative n=109). The results are given as R value (left of slash) with the corresponding p-value (right of slash). **Table 5.** Spearman correlation of the established RA sera of the Predict cohort (n=94; RF IgM and/or α-CCP2 positive n=64, RF IgM and α-CCP2 negative n=30). The results are given as R value (left of slash) with the corresponding p-value (right of slash). **Table 6.** ROC analysis of native hnRNP-DL_mir_ of MTX-treated EIRA patients (n=192; seropositive n=93, seronegative n=99). **Table 7.** Negative CN_DL_-index of MTX-treated EIRA patients n=192 (Resp. n=161, non-Resp. n=31). **Table 8.** ROC analysis of native hnRNP-DL_mir_ of Enbrel®-treated Predict patients (n=94; seropositive n=63, seronegative n=31). **Figure 4.** High baseline titer against α-hnRNP-DL_mir_ (DL) is rather present in 6-month EULAR Responder RA patients who had received MTX or α-TNF inhibitor therapy (Enbrel®). A-C, Citrullinated α-hnRNP-DL_mir_ (citDL) (A), α-hnRNP-DL_mir_ (DL) (B) and ∆ OD between citDL and DL (ΔDL) (C) were measured by ELISA in patient sera from the EIRA cohort treated with MTX (n=192) with 161 EULAR Responder and 31 EULAR non-Responder among 6 months. The evaluation was done according to the cutoff versus other diseases. D, α-DL were measured by ELISA in patient sera from the Predict cohort treated with α-TNF inhibitor therapy with 6-month EULAR response data (n=94, responder n=63, non-Responder n=31). Based on the signals, a response-cutoff (dotted line, OD 0.174) was determined, from which only responders are recognized as positive. OD, optical density; nm, nano meter; RA, rheumatoid arthritis; SLE, systemic lupus erythematosus; MTX, Methotrexate; Resp., 6-month EULAR Responder. **Figure 5.** A, Influence of cytokines on hnRNP-DL expression determined by immunoblotting. Cellular extracts from unstimulated, IL1α- or TNFα-stimulated HeLa cells and from unstimulated and IL6-stimulated HepG2 cells were probed with α-hnRNP-DL1/2-peptide specific rabbit serum. B, Citrullination of hnRNP-DL in synovial tissue from a patient with rheumatoid arthritis was investigated with an α-deiminated arginine antibody and an α-hnRNP-DL antibody. Both positive bands were labled with hnRNP-DL, which isoforms were not analysed.

**Additional file 2: Tab. 1.** Antigens.

## Data Availability

Not applicable

## Abbreviations

AA: Amino acid
ANA HEp-2: Indirect immunofluorescence to anti-nuclear autoantibodies with fixed human epithelial cells
AREs: AU-rich elements
ATXN: Ataxin-2
IL-1Ra: Interleukin-1 receptor antagonist
AUF1: TNFα regulatory protein
BSA: Bovine serum albumin
cit-DL: Citrullinated hnRNP-DL
CN_DL_-Index: Difference of ELISA signal of α-citrullinated and α-native hnRNP-DL (ΔhnRNP-DL)
DTT: Dithiothreitol
ELISA: Enzyme-linked immunosorbent assay
HAM/TSP: HTLV-1-associated myelopathy/tropical spastic paraparesis
HC: Healthy controls
HeLa: Human immortal cell line from cervical cancer taken from Henrietta Lacks
HepG2: A human liver cancer cell line
hnRNP-DL: Heterogeneous nuclear ribonucleoprotein D-like
IL: Interleukin
JKTBP: JKT41-binding protein
LB medium: Lysogeny broth
MB: Ankylosing spondylitis
MCTD: Mixed connective tissue disease
mir: Major immunogenic region
MS: Multiple sclerosis
MTX: Methotrexate
MyD88: Myeloid differentiation primary response gene 88
OA: Osteoarthritis
PBS: Phosphate-buffered saline
PC: Parenchymal changes in the lung
PCR: Polymerase chain reaction
PsA: Psoriasis arthritis
PTMs: Posttranslational modifications
R848: SIGIRR TLR7/8 agonist
RA: Rheumatoid arthritis
RA33: Autoantibodies against hnRNP-A2/B1
RBD: RNA binding domains
RCK/p54: DEAD box/RNA helicase protein
ROC: Receiver operating characteristic
SCE: Structural citrullination dependent epitopes
SDS-PAGE: Sodiumdodecyl sulfate-polyacrylamide electrophoresis
SE: Shared epitope
SIGIRR/TIR8: Single Ig IL-1-related receptor/ Toll/interleukin-1 receptor 8
SLE: Systemic lupus erythematosus
TBS: Tris buffered saline
TBST: Tris buffered saline-Tween
TBST-T: Tris buffered saline-Tween-TritonX
TLR: Toll-like receptor
TNF: Tumour necrosis factor
