# Correction to: The citrullinated/native index of autoantibodies against hnRNP-DL predicts an individual “window of treatment success” in RA patients

**DOI:** 10.1186/s13075-021-02639-z

**Published:** 2021-10-09

**Authors:** Bianka Marklein, Madeleine Jenning, Zoltán Konthur, Thomas Häupl, Franziska Welzel, Ute Nonhoff, Sylvia Krobitsch, Debbie M. Mulder, Marije I. Koenders, Vijay Joshua, Andrew P. Cope, Mark J. Shlomchik, Hans-Joachim Anders, Gerd R. Burmester, Aase Hensvold, Anca I. Catrina, Johan Rönnelid, Günter Steiner, Karl Skriner

**Affiliations:** 1 Department of Rheumatology and Clinical Immunology, Charité — Universitätsmedizin Berlin, Charite Campus Mitte, Rheumatologisches Forschungslabor - AG Skriner, Chariteplatz 1 (intern Virchowweg 11, 5.OG, R011), 10117 Berlin, Germany; 2 German Rheumatism Research Centre, Leibniz Institute, 10117 Berlin, Germany; 3 Max Planck Institute for Molecular Genetics, Berlin, Germany; 4 Max Planck Institute of Colloids and Interfaces, Potsdam, Germany; 5 Department of Analytical Chemistry (Dpt.1), Bundesanstalt für Materialforschung und-prüfung (BAM), Berlin, Germany; 6 Department of Experimental Rheumatology, Radboud University Medical Center, Nijmegen, The Netherlands; 7 Division of Rheumatology, Department of Medicine Solna, Karolinska Institutet, Karolinska University Hospital, Stockholm, Sweden; 8 Centre for Rheumatic Diseases, School of Immunology and Microbial Sciences, Faculty of Life Sciences and Medicine, King’s College London, London, UK; 9 Department of Immunology, University of Pittsburgh School of Medicine, Pittsburgh, PA USA; 10 Medical Clinic and Policlinic IV, Nephrological Center, Ludwig-Maximilian-University Hospital, Munich, Germany; 11 Academic Specialist Center, Center for Rheumatology, Stockholm Health Region, Stockholm, Sweden; 12 Department of Immunology, Genetics and Pathology, Uppsala University, Uppsala, Sweden; 13 Division of Rheumatology, Medical University of Vienna, Vienna, Austria; 14 Ludwig Boltzmann Cluster for Arthritis and Rehabilitation, Vienna, Austria

**Correction to: Arthritis Res Ther 23, 239 (2021)**

**https://doi.org/10.1186/s13075-021-02603-x**

Following publication of the original article [1], the authors reported an error in Additional file 1 wherein the track changes are visible. The Additional file 1 has been updated.

The original article [1] has been updated.

## Supplementary Information

**Additional file 1:** ﻿**Figure 1.** Sequence, structure and major immunogenic region (mir) of hnRNP-D and hnRNP-DL. A, Schematic representation of hnRNP-D (isoform p45), hnRNP-DL and the different recombinant hnRNP-DL variants studied. The main structural features are highlighted. Mir-region is the major immunogenic region, RBD1 and RBD2 are RNA-binding domains 1 and 2, Gly-rich is the C-terminal glycine-rich region of the proteins. B, Global amino acid sequence alignment of hnRNP-D and hnRNP-DL1 (isoform 1). HnRNP-D and -DL share 89.1% similarity by sequenc e[1]. Regions “mir”, “RBD1”, “RBD2” and “Gly-rich” are highlighted. **Figure 2.** Characterisation of autoantibodies against, A, citrullinated α-hnRNP-DLmir (cit-DL), B, α-hnRNP-DLmir (DL) and C, ΔOD between cit-DL and DL (ΔDL) determed by ELISA in sera of other diseases (n=127; MS n=20, reA n=7, Sclero n=20, Sjö n=20, PsA n=20, MB n=20, OA n=20). The dotted lines markes the cutoff vs. other diseases (except systemic lupus erythematosus) or healthy controls with 98% specificity each. OD, optical density; nm, nano meter; vs., versus; MS, multiple sclerosis; reA, reactive arthritis; Sclero, scleroderma; Sjö, Sjögren´s syndrome; PsA, psoriasis arthritis; MB, ankylosing spondylitis; OA. Osteoarthritis. **Table 1.** Mann Whitney U-test of (cit) α-hnRNP-DLmir-OD signals of seropositive and seronegative data sets of RA-cohorts. **Table 2.** Mann Whitney U-test of cit α-hnRNP-DLmir-OD signals of seronegative data sets of RA-cohorts and data sets of other inflammatory diseases. **Figure 3.** XY-Plot and Spearman Correlation of citrullinated or native α-hnRNP-DLmir versus ΔhnRNP-DLmir for the early RA cohort EIRA (A/D; n=404), the seropositive EIRA sera (B/E; n=202) and the seronegative EIRA sera (C/F; n=202). **Table 3.** Spearman correlation of the early RA sera of the EIRA cohort (n=404). The results are given as R value (left of slash) with the corresponding p-value (right of slash). **Table 4.** Spearman correlation of the 242 EIRA sera treated with MTX (α-CCP2 positive n=133, α-CCP2 negative n=109). The results are given as R value (left of slash) with the corresponding p-value (right of slash). **Table 5.** Spearman correlation of the established RA sera of the Predict cohort (n=94; RF IgM and/or α-CCP2 positive n=64, RF IgM and α-CCP2 negative n=30). The results are given as R value (left of slash) with the corresponding p-value (right of slash). **Table 6.** ROC analysis of native hnRNP-DLmir of MTX-treated EIRA patients (n=192; seropositive n=93, seronegative n=99). **Table 7.** Negative CNDL-index of MTX-treated EIRA patients n=192 (Resp. n=161, non-Resp. n=31). **Table 8.** ROC analysis of native hnRNP-DLmir of Enbrel®-treated Predict patients (n=94; seropositive n=63, seronegative n=31). **Figure 4.** High baseline titer against α-hnRNP-DLmir (DL) is rather present in 6-month EULAR Responder RA patients who had received MTX or α-TNF inhibitor therapy (Enbrel®). A-C, Citrullinated α-hnRNP-DLmir (citDL) (A), α-hnRNP-DLmir (DL) (B) and Δ OD between citDL and DL (ΔDL) (C) were measured by ELISA in patient sera from the EIRA cohort treated with MTX (n=192) with 161 EULAR Responder and 31 EULAR non-Responder among 6 months. The evaluation was done according to the cutoff versus other diseases. D, α-DL were measured by ELISA in patient sera from the Predict cohort treated with α-TNF inhibitor therapy with 6-month EULAR response data (n=94, responder n=63, non-Responder n=31). Based on the signals, a response-cutoff (dotted line, OD 0.174) was determined, from which only responders are recognized as positive. OD, optical density; nm, nano meter; RA, rheumatoid arthritis; SLE, systemic lupus erythematosus; MTX, Methotrexate; Resp., 6-month EULAR Responder. **Figure 5.** A, Influence of cytokines on hnRNP-DL expression determined by immunoblotting. Cellular extracts from unstimulated, IL1α- or TNFα-stimulated HeLa cells and from unstimulated and IL6-stimulated HepG2 cells were probed with α-hnRNP-DL1/2-peptide specific rabbit serum. B, Citrullination of hnRNP-DL in synovial tissue from a patient with rheumatoid arthritis was investigated with an α-deiminated arginine antibody and an α-hnRNP-DL antibody. Both positive bands were labled with hnRNP-DL, which isoforms were not analysed.

## References

[1] Marklein B, Jenning M, Konthur Z (2021). The citrullinated/native index of autoantibodies against hnRNP-DL predicts an individual “window of treatment success” in RA patients. Arthritis Res Ther.

