# Supplementary material for: The citrullinated/native index of autoantibodies against hnRNP-DL predicts an individual “window of treatment success” in RA patients
Source: Arthritis Res Ther. 2021 Sep 14;23:239. doi: 10.1186/s13075-021-02603-x (PMC8439038; doi:10.1186/s13075-021-02603-x)
Supplement: Supplementary file 1 — Additional file 1: Figure 1. Sequence, structure and major immunogenic region (mir) of hnRNP-D and hnRNP-DL. A, Schematic representation of hnRNP-D (isoform p45), hnRNP-DL and the different recombinant hnRNP-DL variants studied. The main structural features are highlighted. Mir-region is the major immunogenic region, RBD1 and RBD2 are RNA-binding domains 1 and 2, Gly-rich is the C-terminal glycine-rich region of the proteins. B, Global amino acid sequence alignment of hnRNP-D and hnRNP-DL1 (isoform 1). HnRNP-D and -DL share 89.1% similarity by sequenc e[1]. Regions “mir”, “RBD1”, “RBD2” and “Gly-rich” are highlighted. Figure 2. Characterisation of autoantibodies against, A, citrullinated α-hnRNP-DLmir (cit-DL), B, α-hnRNP-DLmir (DL) and C, ∆OD between cit-DL and DL (ΔDL) determed by ELISA in sera of other diseases (n=127; MS n=20, reA n=7, Sclero n=20, Sjö n=20, PsA n=20, MB n=20, OA n=20). The dotted lines markes the cutoff vs. other diseases (except systemic lupus erythematosus) or healthy controls with 98% specificity each. OD, optical density; nm, nano meter; vs., versus; MS, multiple sclerosis; reA, reactive arthritis; Sclero, scleroderma; Sjö, Sjögren´s syndrome; PsA, psoriasis arthritis; MB, ankylosing spondylitis; OA. Osteoarthritis. Table 1. Mann Whitney U-test of (cit) α-hnRNP-DLmir-OD signals of seropositive and seronegative data sets of RA-cohorts. Table 2. Mann Whitney U-test of cit α-hnRNP-DLmir-OD signals of seronegative data sets of RA-cohorts and data sets of other inflammatory diseases. Figure 3. XY-Plot and Spearman Correlation of citrullinated or native α-hnRNP-DLmir versus ΔhnRNP-DLmir for the early RA cohort EIRA (A/D; n=404), the seropositive EIRA sera (B/E; n=202) and the seronegative EIRA sera (C/F; n=202). Table 3. Spearman correlation of the early RA sera of the EIRA cohort (n=404). The results are given as R value (left of slash) with the corresponding p-value (right of slash). Table 4. Spearman correlation of the 242 EIRA sera treated wi [file 13075_2021_2603_MOESM1_ESM.docx]

**SUPPLEMENTARY RESULTS**

**
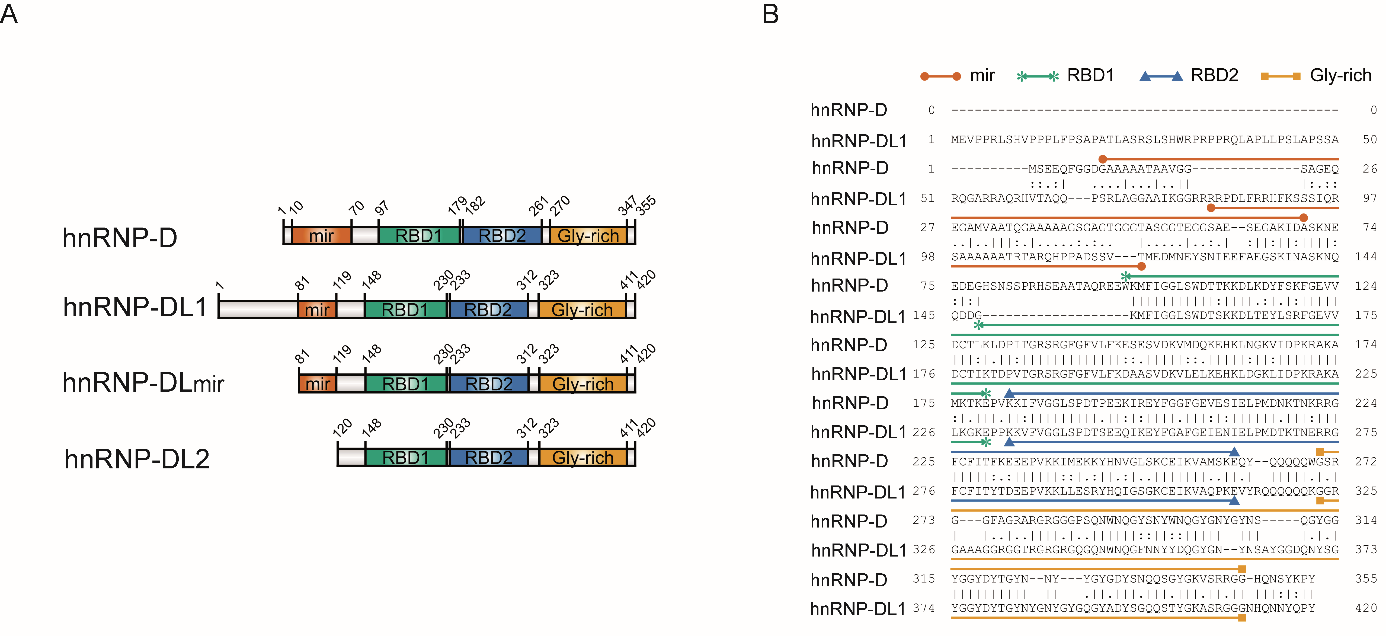
**

**Figure 1**. Sequence, structure and major immunogenic region (mir) of hnRNP-D and hnRNP-DL. **A,** Schematic representation of hnRNP-D (isoform p45), hnRNP-DL and the different recombinant hnRNP-DL variants studied. The main structural features are highlighted. Mir-region is the major immunogenic region, RBD1 and RBD2 are RNA-binding domains 1 and 2, Gly-rich is the C-terminal glycine-rich region of the proteins. **B,** Global amino acid sequence alignment of hnRNP-D and hnRNP-DL1 (isoform 1). HnRNP-D and -DL share 89.1% similarity by sequence[1]. Regions “mir”, “RBD1”, “RBD2” and “Gly-rich” are highlighted.

**
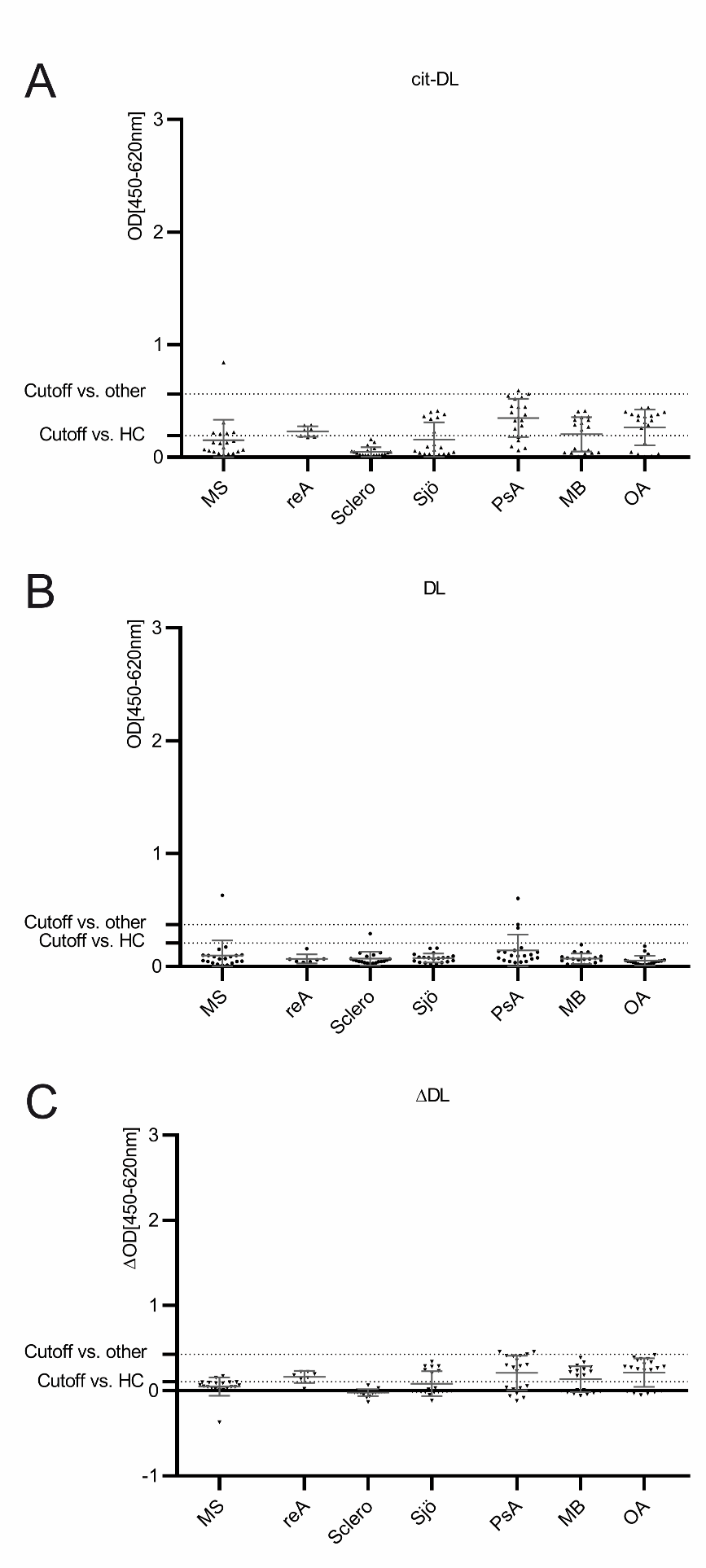
**

**Figure 2**. Characterisation of autoantibodies against, **A**, citrullinated α-hnRNP-DL_mir_ (cit-DL), **B**, α-hnRNP-DL_mir_ (DL) and **C**, ∆OD between cit-DL and DL (ΔDL) determed by ELISA in sera of other diseases (n=127; MS n=20, reA n=7, Sclero n=20, Sjö n=20, PsA n=20, MB n=20, OA n=20). The dotted lines markes the cutoff vs. other diseases (except systemic lupus erythematosus) or healthy controls with 98% specificity each. OD, optical density; nm, nano meter; vs., versus; MS, multiple sclerosis; reA, reactive arthritis; Sclero, scleroderma; Sjö, Sjögren´s syndrome; PsA, psoriasis arthritis; MB, ankylosing spondylitis; OA. Osteoarthritis

**Table 1**. Mann Whitney U-test of (cit) α-hnRNP-DL_mir_-OD signals of seropositive and seronegative data sets of RA-cohorts.

| **cohort** | **Mann Whitney U Test** | **cit-DL** | | **DL** | |
| --- | --- | --- | --- | --- | --- |
|  |  | seropos. | seroneg. | seropos. | seroneg. |
|  |  |  |  |  |  |
|  |  |  |  |  |  |
| **LURA** | median | 0.5480 n=81 | 0.1120 n=25 | 0.06600 n=81 | 0.05700 n=25 |
|  | P value | <0.0001 | | 0.6801 | |
|  | P value summary | **** | | ns | |
|  |  |  |  |  |  |
| **EIRA** | median | 0.7175 n=202 | 0.2403 n=202 | 0.1105 n=202 | 0.1200 n=202 |
|  | P value | <0.0001 | | 0.2672 | |
|  | P value summary | **** | | ns | |
|  |  |  |  |  |  |
| **Predict** | median | 0.8478 n=86 | 0.5255 n=41 | 0.07650 n=86 | 0.06500 n=41 |
|  | P value | <0.0001 | | 0.2693 | |
|  | P value summary | **** | | ns | |

**Table 2**. Mann Whitney U-test of cit α-hnRNP-DL_mir_-OD signals of seronegative data sets of RA-cohorts and data sets of other inflammatory diseases.

| **Mann Whitney Test** | **Cit-DL** | |
| --- | --- | --- |
|  | **seroneg. EIRA** | **other diseases** |
| median | 0.2403 n=202 | 0.1455 n=127 |
| P value | 0.0005 | |
| P value summary | *** | |
|  |  | |
|  | **seroneg. Predict** | **other diseases** |
| median | 0.5255 n=41 | 0.1455 n=127 |
| P value | <0.0001 | |
| P value summary | **** | |
|  |  | |
|  | **seroneg. LURA** | **other diseases** |
| median | 0.1120 n=25 | 0.1455 n=127 |
| P value | 0.2168 | |
| P value summary | ns | |

A

B

C

D

E

F

**Figure 3**. XY-Plot and Spearman Correlation of citrullinated or native α-hnRNP-DL_mir_ versus ΔhnRNP-DL_mir_ for the early RA cohort EIRA (A/D; n=404), the seropositive EIRA sera (B/E; n=202) and the seronegative EIRA sera (C/F; n=202).

**Table 3**. Spearman correlation of the early RA sera of the EIRA cohort (n=404). The results are given as R value (left of slash) with the corresponding p-value (right of slash).

cit-DL, α-citrullinated hnRNP-DL_mir_; DL, α-hnRNP-DL_mir_; ΔDL, difference between optical density of cit-DL and DL; RA, rheumatoid arthritis; RF, rheumatoid factor; CCP2, second generation anti-cyclic citrullinated peptide test; CRP, C-Reactive Protein; ESR, erythrocyte sedimentation rate; SJC, swollen joint count; TJC, tender joint count; VAS, visual analog scale; HAQ, Health Assessment Questionnaire Disability Index; DAS28, disease activity score-28; symptdur, symptom duration; seopos., RF IgM and/or α-CCP2 positive; seroneg., RF IgM and α-CCP2 negative; NS, not significant

**Table 4**. Spearman correlation of the 242 EIRA sera treated with MTX (α-CCP2 positive n=133, α-CCP2 negative n=109). The results are given as R value (left of slash) with the corresponding p-value (right of slash).

cit-DL, α-citrullinated hnRNP-DL_mir_; DL, α-hnRNP-DL_mir_; ΔDL, difference between optical density of cit-DL and DL; RA, rheumatoid arthritis; RF, rheumatoid factor; CCP2, second generation anti-cyclic citrullinated peptide test; CRP, C-Reactive Protein; ESR, erythrocyte sedimentation rate; SJC, swollen joint count; TJC, tender joint count; VAS, visual analog scale; HAQ, Health Assessment Questionnaire Disability Index; DAS28, disease activity score-28; symptdur, symptom duration; seopos., RF IgM and/or α-CCP2 positive; seroneg., RF IgM and α-CCP2 negative; NS, not significant

**Table 5**. Spearman correlation of the established RA sera of the Predict cohort (n=94; RF IgM and/or α-CCP2 positive n=64, RF IgM and α-CCP2 negative n=30). The results are given as R value (left of slash) with the corresponding p-value (right of slash).

cit-DL, α-citrullinated hnRNP-DL_mir_; DL, α-hnRNP-DL_mir_; ΔDL, difference between optical density of cit-DL and DL; RA, rheumatoid arthritis; RF, rheumatoid factor; CCP2, second generation anti-cyclic citrullinated peptide test; CRP, C-Reactive Protein; ESR, erythrocyte sedimentation rate; SJC, swollen joint count; TJC, tender joint count; VAS, visual analog scale; DAS28, disease activity score-28; symptdur, symptom duration; seopos., RF IgM and/or α-CCP2 positive; seroneg., RF IgM and α-CCP2 negative; NS, not significant

**Table 6.** ROC analysis of native hnRNP-DL_mir_ of MTX-treated EIRA patients (n=192; seropositive n=93, seronegative n=99)

**Total n=192**

| Area under the ROC curve |  |
| --- | --- |
| Area | 0.5893 |
| Std. Error | 0.05318 |
| 95% confidence interval | 0.4850 to 0.6935 |
| P value | 0.1159 |
|  |  |
| Data |  |
| Controls (DL_nonR) | 31 |
| Patients (DL_R) | 161 |

|  | **Sensitivity %** | **95% Cl** | **Specificity %** | **95% CI** | **Likelihood Ratio** |
| --- | --- | --- | --- | --- | --- |
| > 0.1540 | 42.86 | 35.47% to 50.58% | 80.65 | 63.72% to 90.81% | 2.214 |
| > 0.1588 | 42.24 | 34.87% to 49.96% | 80.65 | 63.72% to 90.81% | 2.182 |
| > 0.1630 | 41.61 | 34.28% to 49.34% | 80.65 | 63.72% to 90.81% | 2.150 |
| > 0.1650 | 40.99 | 33.69% to 48.71% | 80.65 | 63.72% to 90.81% | 2.118 |
| > 0.1678 | 40.37 | 33.10% to 48.09% | 80.65 | 63.72% to 90.81% | 2.086 |
| > 0.1703 | 39.75 | 32.52% to 47.46% | 80.65 | 63.72% to 90.81% | 2.054 |
| > 0.1718 | 39.13 | 31.93% to 46.84% | 80.65 | 63.72% to 90.81% | 2.022 |
| > 0.1730 | 38.51 | 31.34% to 46.21% | 80.65 | 63.72% to 90.81% | 1.990 |
| > 0.1748 | 37.89 | 30.76% to 45.58% | 80.65 | 63.72% to 90.81% | 1.958 |
| > 0.1758 | 37.27 | 30.18% to 44.95% | 80.65 | 63.72% to 90.81% | 1.925 |
| > 0.1768 | 36.65 | 29.60% to 44.32% | 80.65 | 63.72% to 90.81% | 1.893 |
| > 0.1795 | 36.02 | 29.01% to 43.69% | 80.65 | 63.72% to 90.81% | 1.861 |
| > 0.1830 | 35.40 | 28.44% to 43.05% | 80.65 | 63.72% to 90.81% | 1.829 |
| > 0.1865 | 34.78 | 27.86% to 42.42% | 80.65 | 63.72% to 90.81% | 1.797 |
| > 0.1935 | 34.16 | 27.28% to 41.78% | 80.65 | 63.72% to 90.81% | 1.765 |
| > 0.1998 | 33.54 | 26.71% to 41.14% | 80.65 | 63.72% to 90.81% | 1.733 |
| > 0.2020 | 32.92 | 26.13% to 40.50% | 80.65 | 63.72% to 90.81% | 1.701 |
| > 0.2038 | 32.30 | 25.56% to 39.86% | 80.65 | 63.72% to 90.81% | 1.669 |
| > 0.2048 | 32.30 | 25.56% to 39.86% | 83.87 | 67.37% to 92.91% | 2.002 |
| > 0.2058 | 31.68 | 24.99% to 39.22% | 83.87 | 67.37% to 92.91% | 1.964 |
| **> 0.2068** | **30.43** | **23.85% to 37.93%** | **83.87** | **67.37% to 92.91%** | **1.887** |
| > 0.2078 | 29.81 | 23.29% to 37.28% | 83.87 | 67.37% to 92.91% | 1.848 |
| > 0.2088 | 29.19 | 22.72% to 36.63% | 83.87 | 67.37% to 92.91% | 1.810 |
| > 0.2103 | 28.57 | 22.16% to 35.99% | 83.87 | 67.37% to 92.91% | 1.771 |
| > 0.2120 | 27.95 | 21.59% to 35.33% | 83.87 | 67.37% to 92.91% | 1.733 |
| > 0.2158 | 27.33 | 21.03% to 34.68% | 83.87 | 67.37% to 92.91% | 1.694 |
| > 0.2200 | 26.71 | 20.48% to 34.03% | 83.87 | 67.37% to 92.91% | 1.656 |
| > 0.2215 | 26.09 | 19.92% to 33.37% | 83.87 | 67.37% to 92.91% | 1.617 |
| > 0.2283 | 25.47 | 19.36% to 32.71% | 83.87 | 67.37% to 92.91% | 1.579 |
| > 0.2380 | 24.22 | 18.26% to 31.39% | 83.87 | 67.37% to 92.91% | 1.502 |
| > 0.2428 | 23.60 | 17.71% to 30.73% | 83.87 | 67.37% to 92.91% | 1.463 |
| > 0.2455 | 22.98 | 17.16% to 30.06% | 83.87 | 67.37% to 92.91% | 1.425 |
| > 0.2480 | 22.36 | 16.61% to 29.40% | 83.87 | 67.37% to 92.91% | 1.386 |
| > 0.2498 | 22.36 | 16.61% to 29.40% | 87.10 | 71.15% to 94.87% | 1.733 |
| > 0.2513 | 22.36 | 16.61% to 29.40% | 90.32 | 75.10% to 96.65% | 2.311 |
| > 0.2608 | 21.74 | 16.07% to 28.73% | 90.32 | 75.10% to 96.65% | 2.246 |
| > 0.2703 | 21.12 | 15.52% to 28.06% | 90.32 | 75.10% to 96.65% | 2.182 |
| > 0.2740 | 20.50 | 14.98% to 27.39% | 90.32 | 75.10% to 96.65% | 2.118 |
| > 0.2780 | 19.88 | 14.45% to 26.71% | 90.32 | 75.10% to 96.65% | 2.054 |
| > 0.2873 | 19.25 | 13.91% to 26.03% | 90.32 | 75.10% to 96.65% | 1.990 |
| > 0.2983 | 18.63 | 13.38% to 25.35% | 90.32 | 75.10% to 96.65% | 1.925 |
| > 0.3028 | 18.01 | 12.84% to 24.67% | 90.32 | 75.10% to 96.65% | 1.861 |
| > 0.3148 | 17.39 | 12.32% to 23.99% | 90.32 | 75.10% to 96.65% | 1.797 |
| > 0.3285 | 16.77 | 11.79% to 23.30% | 90.32 | 75.10% to 96.65% | 1.733 |
| > 0.3323 | 16.15 | 11.27% to 22.61% | 90.32 | 75.10% to 96.65% | 1.669 |
| > 0.3328 | 15.53 | 10.74% to 21.92% | 90.32 | 75.10% to 96.65% | 1.605 |
| > 0.3378 | 14.91 | 10.23% to 21.22% | 90.32 | 75.10% to 96.65% | 1.540 |
| > 0.3430 | 14.29 | 9.712% to 20.52% | 90.32 | 75.10% to 96.65% | 1.476 |
| > 0.3445 | 13.66 | 9.200% to 19.82% | 90.32 | 75.10% to 96.65% | 1.412 |
| > 0.3555 | 13.04 | 8.692% to 19.12% | 90.32 | 75.10% to 96.65% | 1.348 |
| > 0.3683 | 12.42 | 8.187% to 18.41% | 90.32 | 75.10% to 96.65% | 1.284 |
| **> 0.3713** | **11.80** | **7.687% to 17.70%** | **90.32** | **75.10% to 96.65%** | **1.219** |
| > 0.3800 | 11.18 | 7.190% to 16.98% | 90.32 | 75.10% to 96.65% | 1.155 |
| > 0.3985 | 10.56 | 6.698% to 16.26% | 90.32 | 75.10% to 96.65% | 1.091 |
| > 0.4133 | 9.938 | 6.210% to 15.53% | 90.32 | 75.10% to 96.65% | 1.027 |
| > 0.4193 | 9.317 | 5.727% to 14.80% | 90.32 | 75.10% to 96.65% | 0.9627 |
| > 0.4605 | 8.696 | 5.250% to 14.07% | 90.32 | 75.10% to 96.65% | 0.8986 |
| > 0.5048 | 8.075 | 4.779% to 13.32% | 90.32 | 75.10% to 96.65% | 0.8344 |
| > 0.5175 | 7.453 | 4.315% to 12.58% | 90.32 | 75.10% to 96.65% | 0.7702 |
| > 0.5358 | 7.453 | 4.315% to 12.58% | 93.55 | 79.28% to 98.85% | 1.155 |
| > 0.5495 | 6.832 | 3.858% to 11.82% | 93.55 | 79.28% to 98.85% | 1.059 |
| > 0.5663 | 6.211 | 3.408% to 11.05% | 93.55 | 79.28% to 98.85% | 0.9627 |
| > 0.6030 | 5.590 | 2.969% to 10.28% | 93.55 | 79.28% to 98.85% | 0.8665 |
| > 0.6288 | 5.590 | 2.969% to 10.28% | 96.77 | 83.81% to 99.83% | 1.733 |
| > 0.6663 | 4.969 | 2.539% to 9.498% | 96.77 | 83.81% to 99.83% | 1.540 |
| > 0.7028 | 4.348 | 2.122% to 8.702% | 96.77 | 83.81% to 99.83% | 1.348 |
| > 0.7125 | 3.727 | 1.719% to 7.891% | 96.77 | 83.81% to 99.83% | 1.155 |
| > 0.7370 | 3.106 | 1.334% to 7.063% | 96.77 | 83.81% to 99.83% | 0.9627 |
| > 0.7533 | 2.484 | 0.9703% to 6.213% | 96.77 | 83.81% to 99.83% | 0.7702 |
| > 0.7605 | 1.863 | 0.5079% to 5.335% | 96.77 | 83.81% to 99.83% | 0.5776 |
| > 0.8710 | 1.242 | 0.2207% to 4.416% | 96.77 | 83.81% to 99.83% | 0.3851 |
| > 1.538 | 0.6211 | 0.03186% to 3.434% | 96.77 | 83.81% to 99.83% | 0.1925 |
| > 2.102 | 0.000 | 0.000% to 2.330% | 96.77 | 83.81% to 99.83% | 0.000 |

**seropositive n=93**

| Area under the ROC curve |  |
| --- | --- |
| Area | 0.5308 |
| Std. Error | 0.08058 |
| 95% confidence interval | 0.3728 to 0.6887 |
| P value | 0.7069 |
|  |  |
| Data |  |
| Controls (DL_seropos_nonR) | 15 |
| Patients (DL_seropos_R) | 78 |

|  | **Sensitivity %** | **95% Cl** | **Specificity %** | **95% CI** | **Likelihood Ratio** |
| --- | --- | --- | --- | --- | --- |
| > 0.2058 | 29.49 | 20.52% to 40.38% | 80.00 | 54.81% to 92.95% | 1.474 |
| **> 0.2078** | **28.21** | **19.43% to 39.03%** | **80.00** | **54.81% to 92.95%** | **1.410** |
| > 0.2088 | 26.92 | 18.34% to 37.68% | 80.00 | 54.81% to 92.95% | 1.346 |
| > 0.2103 | 25.64 | 17.26% to 36.31% | 80.00 | 54.81% to 92.95% | 1.282 |
| > 0.2153 | 24.36 | 16.19% to 34.94% | 80.00 | 54.81% to 92.95% | 1.218 |
| > 0.2205 | 23.08 | 15.13% to 33.56% | 80.00 | 54.81% to 92.95% | 1.154 |
| > 0.2283 | 21.79 | 14.08% to 32.16% | 80.00 | 54.81% to 92.95% | 1.090 |
| > 0.2425 | 19.23 | 12.02% to 29.33% | 80.00 | 54.81% to 92.95% | 0.9615 |
| > 0.2638 | 19.23 | 12.02% to 29.33% | 86.67 | 62.12% to 97.63% | 1.442 |
| > 0.2863 | 17.95 | 11.00% to 27.90% | 86.67 | 62.12% to 97.63% | 1.346 |
| > 0.2983 | 16.67 | 10.01% to 26.46% | 86.67 | 62.12% to 97.63% | 1.250 |
| > 0.3028 | 15.38 | 9.026% to 24.99% | 86.67 | 62.12% to 97.63% | 1.154 |
| > 0.3148 | 14.10 | 8.061% to 23.51% | 86.67 | 62.12% to 97.63% | 1.058 |
| > 0.3285 | 12.82 | 7.115% to 22.02% | 86.67 | 62.12% to 97.63% | 0.9615 |
| > 0.3373 | 11.54 | 6.191% to 20.50% | 86.67 | 62.12% to 97.63% | 0.8654 |
| > 0.3430 | 10.26 | 5.289% to 18.95% | 86.67 | 62.12% to 97.63% | 0.7692 |
| > 0.3445 | 8.974 | 4.415% to 17.38% | 86.67 | 62.12% to 97.63% | 0.6731 |
| **> 0.3770** | **7.692** | **3.573% to 15.78%** | **86.67** | **62.12% to 97.63%** | **0.5769** |
| > 0.4545 | 6.410 | 2.769% to 14.14% | 86.67 | 62.12% to 97.63% | 0.4808 |
| > 0.5230 | 5.128 | 2.012% to 12.46% | 86.67 | 62.12% to 97.63% | 0.3846 |
| > 0.5495 | 3.846 | 1.048% to 10.71% | 86.67 | 62.12% to 97.63% | 0.2885 |
| > 0.5903 | 2.564 | 0.4556% to 8.875% | 86.67 | 62.12% to 97.63% | 0.1923 |
| > 0.6743 | 2.564 | 0.4556% to 8.875% | 93.33 | 70.18% to 99.66% | 0.3846 |
| > 0.8483 | 1.282 | 0.06576% to 6.911% | 93.33 | 70.18% to 99.66% | 0.1923 |
| > 1.540 | 0.000 | 0.000% to 4.694% | 93.33 | 70.18% to 99.66% | 0.000 |

**seronegative n=99**

| Area under the ROC curve |  |
| --- | --- |
| Area | 0.6423 |
| Std. Error | 0.06894 |
| 95% confidence interval | 0.5072 to 0.7774 |
| P value | 0.0724 |
|  |  |
| Data |  |
| Controls (DL_seroneg_nonR) | 16 |
| Patients (DL_seroneg_R) | 83 |

|  | **Sensitivity %** | **95% Cl** | **Specificity %** | **95% CI** | **Likelihood Ratio** |
| --- | --- | --- | --- | --- | --- |
| > 0.1365 | 50.60 | 40.06% to 61.09% | 81.25 | 56.99% to 93.41% | 2.699 |
| > 0.1415 | 49.40 | 38.91% to 59.94% | 81.25 | 56.99% to 93.41% | 2.635 |
| > 0.1458 | 48.19 | 37.76% to 58.78% | 81.25 | 56.99% to 93.41% | 2.570 |
| > 0.1470 | 46.99 | 36.62% to 57.62% | 81.25 | 56.99% to 93.41% | 2.506 |
| > 0.1490 | 45.78 | 35.49% to 56.45% | 81.25 | 56.99% to 93.41% | 2.442 |
| > 0.1508 | 44.58 | 34.36% to 55.27% | 81.25 | 56.99% to 93.41% | 2.378 |
| > 0.1520 | 42.17 | 32.12% to 52.91% | 81.25 | 56.99% to 93.41% | 2.249 |
| > 0.1540 | 42.17 | 32.12% to 52.91% | 87.50 | 63.98% to 97.78% | 3.373 |
| > 0.1608 | 40.96 | 31.01% to 51.71% | 87.50 | 63.98% to 97.78% | 3.277 |
| > 0.1703 | 39.76 | 29.91% to 50.52% | 87.50 | 63.98% to 97.78% | 3.181 |
| > 0.1748 | 38.55 | 28.81% to 49.31% | 87.50 | 63.98% to 97.78% | 3.084 |
| > 0.1785 | 37.35 | 27.72% to 48.10% | 87.50 | 63.98% to 97.78% | 2.988 |
| > 0.1850 | 36.14 | 26.63% to 46.88% | 87.50 | 63.98% to 97.78% | 2.892 |
| > 0.1968 | 34.94 | 25.56% to 45.66% | 87.50 | 63.98% to 97.78% | 2.795 |
| **> 0.2058** | **33.73** | **24.48% to 44.42%** | **87.50** | **63.98% to 97.78%** | **2.699** |
| > 0.2095 | 31.33 | 22.36% to 41.94% | 87.50 | 63.98% to 97.78% | 2.506 |
| > 0.2168 | 30.12 | 21.31% to 40.69% | 87.50 | 63.98% to 97.78% | 2.410 |
| > 0.2313 | 28.92 | 20.27% to 39.43% | 87.50 | 63.98% to 97.78% | 2.313 |
| > 0.2428 | 27.71 | 19.23% to 38.16% | 87.50 | 63.98% to 97.78% | 2.217 |
| > 0.2455 | 26.51 | 18.20% to 36.89% | 87.50 | 63.98% to 97.78% | 2.120 |
| > 0.2480 | 25.30 | 17.19% to 35.60% | 87.50 | 63.98% to 97.78% | 2.024 |
| > 0.2505 | 25.30 | 17.19% to 35.60% | 93.75 | 71.67% to 99.68% | 4.048 |
| > 0.2608 | 24.10 | 16.17% to 34.31% | 93.75 | 71.67% to 99.68% | 3.855 |
| > 0.2703 | 22.89 | 15.17% to 33.01% | 93.75 | 71.67% to 99.68% | 3.663 |
| > 0.2750 | 21.69 | 14.18% to 31.70% | 93.75 | 71.67% to 99.68% | 3.470 |
| > 0.3058 | 20.48 | 13.20% to 30.38% | 93.75 | 71.67% to 99.68% | 3.277 |
| > 0.3328 | 19.28 | 12.23% to 29.04% | 93.75 | 71.67% to 99.68% | 3.084 |
| > 0.3493 | 18.07 | 11.27% to 27.70% | 93.75 | 71.67% to 99.68% | 2.892 |
| > 0.3683 | 16.87 | 10.32% to 26.34% | 93.75 | 71.67% to 99.68% | 2.699 |
| **> 0.3713** | **15.66** | **9.388% to 24.98%** | **93.75** | **71.67% to 99.68%** | **2.506** |
| > 0.3800 | 14.46 | 8.468% to 23.59% | 93.75 | 71.67% to 99.68% | 2.313 |
| > 0.4033 | 13.25 | 7.564% to 22.19% | 93.75 | 71.67% to 99.68% | 2.120 |
| > 0.4193 | 12.05 | 6.678% to 20.78% | 93.75 | 71.67% to 99.68% | 1.928 |
| > 0.4648 | 10.84 | 5.810% to 19.34% | 93.75 | 71.67% to 99.68% | 1.735 |
| > 0.5175 | 9.639 | 4.965% to 17.88% | 93.75 | 71.67% to 99.68% | 1.542 |
| > 0.5525 | 9.639 | 4.965% to 17.88% | 100.0 | 80.64% to 100.0% |  |
| > 0.6048 | 8.434 | 4.145% to 16.40% | 100.0 | 80.64% to 100.0% |  |
| > 0.6663 | 7.229 | 3.355% to 14.89% | 100.0 | 80.64% to 100.0% |  |
| > 0.7028 | 6.024 | 2.600% to 13.34% | 100.0 | 80.64% to 100.0% |  |
| > 0.7280 | 4.819 | 1.890% to 11.75% | 100.0 | 80.64% to 100.0% |  |
| > 0.7533 | 3.614 | 0.9852% to 10.10% | 100.0 | 80.64% to 100.0% |  |
| > 0.7605 | 2.410 | 0.4281% to 8.366% | 100.0 | 80.64% to 100.0% |  |
| > 1.434 | 1.205 | 0.06180% to 6.514% | 100.0 | 80.64% to 100.0% |  |

**Table 7.** Negative CN_DL_-index of MTX-treated EIRA patients n=192 (Resp. n=161, non-Resp. n=31)

| ΔDL_Responder  N=27 | ΔDL_nonResponder  N=4 |
| --- | --- |
| -0.759 | -0.3705 |
| -0.729 | -0.199 |
| -0.5745 | -0.1225 |
| -0.41 | -0.032 |
| -0.3395 |  |
| -0.3 |  |
| -0.27 |  |
| -0.2325 |  |
| -0.2145 |  |
| -0.186 |  |
| -0.184 |  |
| -0.175 |  |
| -0.14 |  |
| -0.118 |  |
| -0.091 |  |
| -0.0835 |  |
| -0.08 |  |
| -0.0765 |  |
| -0.0635 |  |
| -0.0435 |  |
| -0.032 |  |
| -0.024 |  |
| -0.0185 |  |
| -0.0155 |  |
| -0.0145 |  |
| -0.0095 |  |
| -0.007 |  |

Cutoff = -0.2010 🡪33% Sensitivity/ 75% Specificity

Cutoff = -0.3710 🡪15% Sensitivity/ 100% Specificity

**Table 8.** ROC analysis of native hnRNP-DL_mir_ of Enbrel^®^-treated Predict patients (n=94; seropositive n=63, seronegative n=31)

**Total n=192**

| Area under the ROC curve |  |
| --- | --- |
| Area | 0.5246 |
| Std. Error | 0.06190 |
| 95% confidence interval | 0.4033 to 0.6459 |
| P value | 0.6995 |
|  |  |
| Data |  |
| Controls (DL_nonR) | 31 |
| Patients (DL_R) | 63 |

|  | **Sensitivity %** | **95% Cl** | **Specificity %** | **95% CI** | **Likelihood Ratio** |
| --- | --- | --- | --- | --- | --- |
| > 0.1118 | 23.81 | 14.99% to 35.64% | 80.65 | 63.72% to 90.81% | 1.230 |
| > 0.1153 | 22.22 | 13.73% to 33.91% | 80.65 | 63.72% to 90.81% | 1.148 |
| > 0.1178 | 22.22 | 13.73% to 33.91% | 83.87 | 67.37% to 92.91% | 1.378 |
| > 0.1213 | 20.63 | 12.48% to 32.17% | 83.87 | 67.37% to 92.91% | 1.279 |
| > 0.1245 | 19.05 | 11.25% to 30.41% | 83.87 | 67.37% to 92.91% | 1.181 |
| > 0.1270 | 19.05 | 11.25% to 30.41% | 87.10 | 71.15% to 94.87% | 1.476 |
| > 0.1308 | 17.46 | 10.04% to 28.62% | 87.10 | 71.15% to 94.87% | 1.353 |
| > 0.1358 | 17.46 | 10.04% to 28.62% | 90.32 | 75.10% to 96.65% | 1.804 |
| > 0.1443 | 15.87 | 8.857% to 26.81% | 90.32 | 75.10% to 96.65% | 1.640 |
| > 0.1558 | 12.70 | 6.577% to 23.11% | 90.32 | 75.10% to 96.65% | 1.312 |
| > 0.1638 | 12.70 | 6.577% to 23.11% | 93.55 | 79.28% to 98.85% | 1.968 |
| > 0.1703 | 12.70 | 6.577% to 23.11% | 96.77 | 83.81% to 99.83% | 3.937 |
| **> 0.1740** | **12.70** | **6.577% to 23.11%** | **100.0** | **88.97% to 100.0%** |  |
| > 0.1985 | 9.524 | 4.438% to 19.26% | 100.0 | 88.97% to 100.0% |  |
| > 0.2263 | 7.937 | 3.438% to 17.27% | 100.0 | 88.97% to 100.0% |  |
| > 0.2373 | 6.349 | 2.497% to 15.22% | 100.0 | 88.97% to 100.0% |  |
| > 0.2515 | 4.762 | 1.298% to 13.09% | 100.0 | 88.97% to 100.0% |  |
| > 0.2753 | 3.175 | 0.5641% to 10.86% | 100.0 | 88.97% to 100.0% |  |
| > 0.2930 | 1.587 | 0.08142% to 8.459% | 100.0 | 88.97% to 100.0% |  |

**seropositive n=63**

| Area | 0.5785 |
| --- | --- |
| Std. Error | 0.08150 |
| 95% confidence interval | 0.4187 to 0.7382 |
| P value | 0.3515 |
|  |  |
| Data |  |
| Controls (DL_seropos_nonR) | 16 |
| Patients (DL_seropos_R) | 47 |

|  | **Sensitivity %** | **95% Cl** | **Specificity %** | **95% CI** | **Likelihood Ratio** |
| --- | --- | --- | --- | --- | --- |
| < 0.02575 | 2.128 | 0.1091% to 11.11% | 100.0 | 80.64% to 100.0% |  |
| < 0.02625 | 4.255 | 0.7561% to 14.25% | 100.0 | 80.64% to 100.0% |  |
| < 0.02800 | 6.383 | 2.195% to 17.16% | 100.0 | 80.64% to 100.0% |  |
| < 0.03025 | 6.383 | 2.195% to 17.16% | 93.75 | 71.67% to 99.68% | 1.021 |
| < 0.03375 | 8.511 | 3.359% to 19.93% | 93.75 | 71.67% to 99.68% | 1.362 |
| < 0.03700 | 8.511 | 3.359% to 19.93% | 87.50 | 63.98% to 97.78% | 0.6809 |
| < 0.03775 | 12.77 | 5.985% to 25.17% | 87.50 | 63.98% to 97.78% | 1.021 |
| < 0.03850 | 14.89 | 7.407% to 27.69% | 87.50 | 63.98% to 97.78% | 1.191 |
| < 0.03925 | 17.02 | 8.886% to 30.14% | 87.50 | 63.98% to 97.78% | 1.362 |
| < 0.03975 | 19.15 | 10.42% to 32.54% | 87.50 | 63.98% to 97.78% | 1.532 |
| < 0.04225 | 21.28 | 11.99% to 34.90% | 87.50 | 63.98% to 97.78% | 1.702 |
| < 0.04725 | 23.40 | 13.60% to 37.22% | 87.50 | 63.98% to 97.78% | 1.872 |
| < 0.05075 | 25.53 | 15.25% to 39.51% | 87.50 | 63.98% to 97.78% | 2.043 |
| < 0.05200 | 25.53 | 15.25% to 39.51% | 81.25 | 56.99% to 93.41% | 1.362 |
| < 0.05275 | 27.66 | 16.94% to 41.76% | 81.25 | 56.99% to 93.41% | 1.475 |
| < 0.05375 | 31.91 | 20.40% to 46.17% | 81.25 | 56.99% to 93.41% | 1.702 |
| < 0.05475 | 34.04 | 22.17% to 48.33% | 81.25 | 56.99% to 93.41% | 1.816 |

**seronegative n=31**

| Area under the ROC curve |  |
| --- | --- |
| Area | 0.6479 |
| Std. Error | 0.1014 |
| 95% confidence interval | 0.4493 to 0.8466 |
| P value | 0.1605 |
|  |  |
| Data |  |
| Controls (DL_seroneg_nonR) | 15 |
| Patients (DL_seroneg_R) | 16 |

|  | **Sensitivity %** | **95% Cl** | **Specificity %** | **95% CI** | **Likelihood Ratio** |
| --- | --- | --- | --- | --- | --- |
| > 0.08225 | 50.00 | 28.00% to 72.00% | 80.00 | 54.81% to 92.95% | 2.500 |
| > 0.08475 | 43.75 | 23.10% to 66.82% | 80.00 | 54.81% to 92.95% | 2.188 |
| > 0.09550 | 43.75 | 23.10% to 66.82% | 86.67 | 62.12% to 97.63% | 3.281 |
| > 0.1065 | 37.50 | 18.48% to 61.36% | 93.33 | 70.18% to 99.66% | 5.625 |
| > 0.1143 | 31.25 | 14.16% to 55.60% | 93.33 | 70.18% to 99.66% | 4.688 |
| > 0.1233 | 25.00 | 10.18% to 49.50% | 93.33 | 70.18% to 99.66% | 3.750 |
| **> 0.1320** | **25.00** | **10.18% to 49.50%** | **100.0** | **79.61% to 100.0%** |  |
| > 0.1560 | 18.75 | 6.592% to 43.01% | 100.0 | 79.61% to 100.0% |  |
| > 0.1985 | 12.50 | 2.221% to 36.02% | 100.0 | 79.61% to 100.0% |  |
| > 0.2263 | 6.250 | 0.3206% to 28.33% | 100.0 | 79.61% to 100.0% |  |


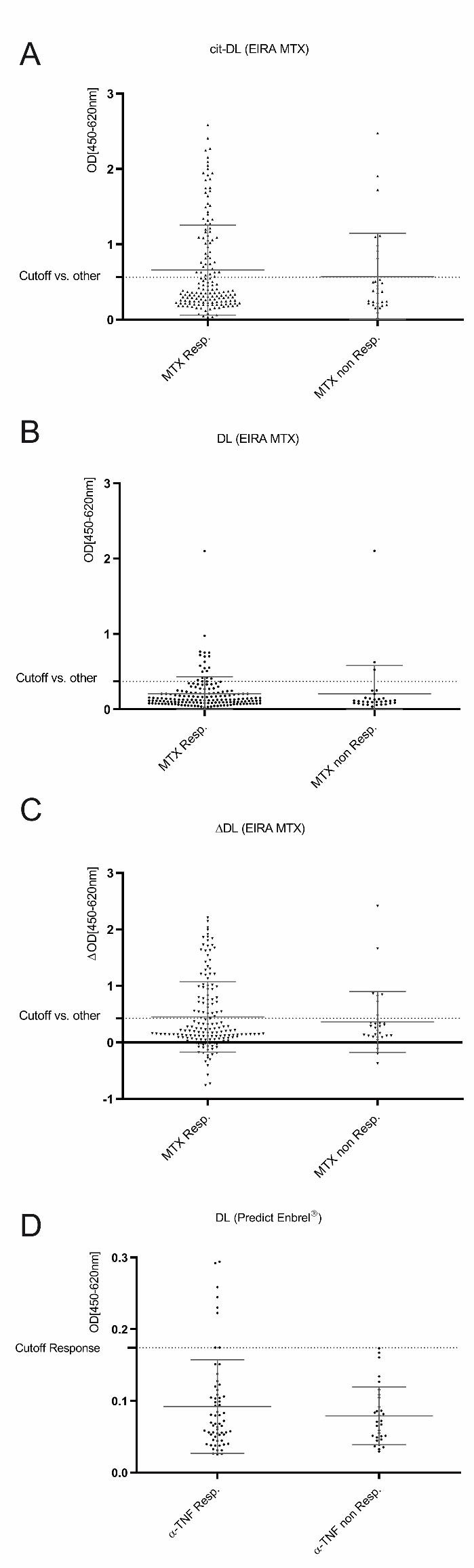


**Figure 4**. High baseline titer against α-hnRNP-DL_mir_ (DL) is rather present in 6-month EULAR Responder RA patients who had received MTX or α-TNF inhibitor therapy (Enbrel^®^). **A-C**, Citrullinated α-hnRNP-DL_mir_ (citDL) (**A**), α-hnRNP-DL_mir_ (DL) (**B**) and ∆ OD between citDL and DL (ΔDL) (**C**) were measured by ELISA in patient sera from the EIRA cohort treated with MTX (n=192) with 161 EULAR Responder and 31 EULAR non-Responder among 6 months. The evaluation was done according to the cutoff versus other diseases. **D**, α-DL were measured by ELISA in patient sera from the Predict cohort treated with α-TNF inhibitor therapy with 6-month EULAR response data (n=94, responder n=63, non-Responder n=31). Based on the signals, a response-cutoff (dotted line, OD 0.174) was determined, from which only responders are recognized as positive.

OD, optical density; nm, nano meter; RA, rheumatoid arthritis; SLE, systemic lupus erythematosus; MTX, Methotrexate; Resp., 6-month EULAR Responder

**
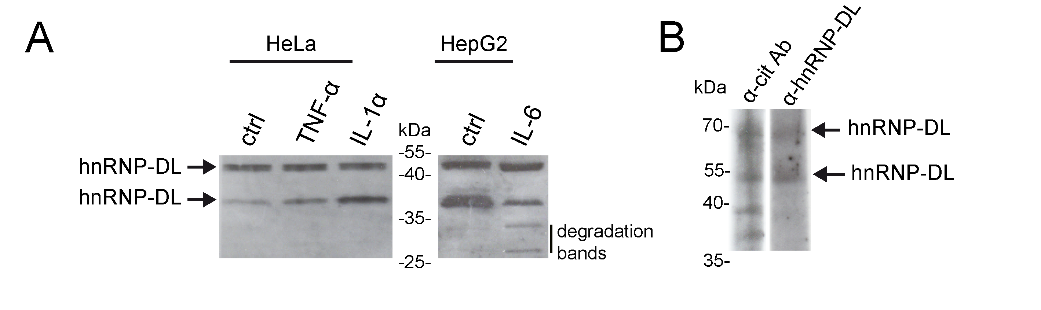
**

**Figure 5**. A, Influence of cytokines on hnRNP-DL expression determined by immunoblotting. Cellular extracts from unstimulated, IL1α- or TNFα-stimulated HeLa cells and from unstimulated and IL6-stimulated HepG2 cells were probed with α-hnRNP-DL1/2-peptide specific rabbit serum. B, Citrullination of hnRNP-DL in synovial tissue from a patient with rheumatoid arthritis was investigated with an α-deiminated arginine antibody and an α-hnRNP-DL antibody. Both positive bands were labled with hnRNP-DL, which isoforms were not analysed.

**REFERENCES**

1. Needleman, S.B. and C.D. Wunsch, *A general method applicable to the search for similarities in the amino acid sequence of two proteins.* J Mol Biol, 1970. **48**(3): p. 443-53.
